# Transcriptomic and metabolomic profiling of melatonin treated soybean (*Glycine max* L.) under drought stress during grain filling period through regulation of secondary metabolite biosynthesis pathways

**DOI:** 10.1371/journal.pone.0239701

**Published:** 2020-10-30

**Authors:** Liang Cao, Xijun Jin, Yuxian Zhang, Mingcong Zhang, Yanhong Wang

**Affiliations:** College of Agronomy, Heilongjiang Bayi Agricultural University, Daqing, Heilongjiang, China; Chinese University of Hong Kong, HONG KONG

## Abstract

There is a growing need to enhance the productivity of soybean (*Glycine max* L.) under severe drought conditions in order to improve global food security status. Melatonin, a ubiquitous hormone, could alleviate drought stress in various plants. Earlier, we demonstrated that exogenous melatonin treatment could enhance the tolerance of drought-treated soybean. However, the underlying mechanisms by which this hormone exerts drought resistance is still unclear. The present study used transcriptomic and metabolomic techniques to determine some critical genes and pathways regulating melatonin response to drought conditions. Results showed that exogenous melatonin treatment could increase relative water content and decrease electrolyte leakage in the leaves and increase seed yield under drought stress. Transcriptomic analysis showed that there were 852 core differentially expressed genes (DEGs) that were regulated by drought stress and melatonin in soybean leaves. The most enriched drought-responsive genes are mainly involved in the ‘biosynthesis of secondary metabolites’. Metabolomic profiling under drought stress showed higher accumulation levels of secondary metabolites related to drought tolerance after exogenous melatonin treatment. Also, we highlighted the vital role of the pathways including phenylpropanoid, flavonoid, isoflavonoid, and steroid biosynthesis pathways for improvement of drought tolerance in soybean by exogenous melatonin treatment. In all, findings from this study give detailed molecular basis for the application of melatonin as a drought-resistant agent in soybean cultivation.

## Introduction

As a significant world cash crop, soybean (*Glycine max* L.) is an essential source of dietary oils and edible proteins [1]. However, soybean production levels is negatively impacted by an array of environmental stressors, the most deleterious of which is arguably drought-related as it affects every developmental stage of the crop [2]. As an abiotic factor, drought can lower agricultural productivity by 50%, and reports from climate models suggest that global warming-triggered drought incidences will be a more regular occurrence [3]. More so, global soybean consumption is expected to increase significantly with the ongoing rise in global human population [4]. This has informed several research initiatives in developing more climate-smart agricultural protocols to ensure food sustainability.

Many Agricultural scientists have recently focused on drought-tolerance mechanisms with which plants induce several defense pathways that make them resilient during drought seasons. These defense pathways could activate various molecular, biochemical, and physiological responses, depending on the type and intensity of the stress signal perception and transduction [5–9]. However, these may be inadequate under highly unfavorable drought stress. Therefore, the use of exogenous protocols has been proposed as an eco-friendly alternative to ensure high soybean productivity levels under drought sessions. Although irrigation is a viable option, it can have its limitations. In the United States, for example, only 9% of soybean-planted lands are irrigated. A more feasible alternative can be the modification of plant hormones to improve plant productivity and lower susceptibility to drought stress.

As a versatile hormone, melatonin (N-acetyl-5-methoxytryptamine) is a highly conserved molecule in plants and animals [10]. Its ubiquity implies that it is of high value to these organisms [11]. Melatonin suppresses the activities of plant stressors such as chemical pollutants, unfavorable temperature ranges, and pathogenic attacks. [12–16]. As an important plant growth regulator, it has also been linked with increasing plant drought tolerance [17–20]. Melatonin could endow plants with drought tolerance features by regulating several complex biological and molecular pathways [21–23]. However, studies on the use of melatonin as an exogenous inducer in improving soybean drought tolerance are scarce.

Previously, we reported that exogenous melatonin treatment could improve the tolerance of drought-treated soybean, which was possibly due to the enhanced content of osmolytes and higher antioxidant enzyme activities that reduced dehydration and lipid peroxidation [24]. However, there has been scarce reports on detailed mechanisms of melatonin for inducing drought resistance in soybean. Significant advancements in omics techniques have resolved many challenges in crop plant research [25–27]. In addition, transcriptomic and metabolomic analyses of drought-induced defense pathways are still poorly understood in soybeans. In this study, the transcriptomic and metabolomic profiling of melatonin for inducing resistance to drought stress in leaves of soybean was carried out, and we analyzed how melatonin response to drought stress by integrated omics strategies. It is anticipated that these findings will provide crucial insights into the theory and application for melatonin as a soybean drought-resistant agent.

## Materials and methods

### Plant materials and experimental design

This research was carried out in 2018 at Heilongjiang Bayi Agricultural University, Northeast China (124°19'-125°12'E, 45°46'-46°55'N). Eight Suinong 26 soybean (*Glycine max* L.) variety seeds were sown per plastic pot (33 × 30 cm) in a 1:1 (v/v) vermiculite and perlite mixture. Watering of pots with Hoagland solution was done once daily from sowing to emergence, and after development, twice daily. Distilled water was applied every 5 d to stop salt and vermiculite/perlite accumulations. Seedlings were collected at the cotyledon stage (VC).

The growth temperature was shown in S1 Table. Planting pots during the grain filling stage (S1 Fig) were divided into well-watered and drought-stressed groups following a previously described method [28]. The former was kept at 80% relative soil water content (RSWC) while the latter was maintained at 45% RSWC. 50% of these pots were treated with 45 mL of a 100 μmol L^-1^ melatonin solution for 3 d [17], while the remaining were sprayed with distilled water as a control treatment. The weight of each pot was taken daily for a period of 10 d to ensure that the set RSWC limits were maintained.

There were three treatments: (1) control (CK): well-watered (80%) and sprayed with distilled water; (2) drought (D): drought-stressed (45%) and sprinkled with distilled water; (3) D-M: drought-stressed (45%) and sprayed with melatonin. A randomized complete design with three replications was adopted for this study. Full-grown leaf samples were collected from each treatment after 3 d, sprayed with melatonin solution and frozen in liquid nitrogen, and stored at -80°C.

### Determination of the relative water content and electrolyte leakage in the leaf and seed yield

The relative water content (RWC) was determined by oven drying method. Briefly, the weight of fresh soybean leaf samples was measured (W1). The leaf at the drum stage was incubated with sterile distilled water for 12 h, and the weight of the leaf was determined after water surface absorbance by filter paper (BW). Finally, it was baked to constant weight to evaluate the dry weight (W2). The following formula was used to determine the RWC: RWC = (W1—W2)/(BW—W2).

The electrolyte leakage (E_L_) was measured on the day of sampling, and the surface of the leaf was washed with distilled water. After cleaning, the leaf was incubated with sterile distilled water for 24 h. The initial conductivity (E_0_) was measured using a conductivity meter. The tube was heated at 120°C in a boiling water bath for 30 min, and the maximum conductivity (E_max_) was determined after cooling. The E_L_ was computed as follow: E_L_ (%) = E_0_/E_max_.

The pod number per plant, number of grains per plant, 100-seed weight, and grain weight per plant at maturity were determined based on 15 representative samples in each group.

### Total RNA isolation and transcriptome analysis

The EASYspin Plus kit (Aidlab, Beijing, China) was used to extract the RNA of 9 samples following the manufacturer’s instructions. A total of 9 RNA-Seq libraries were prepared and sequenced as described recently [29]. The obtained raw data is available in the National Center for Biotechnology Information (NCBI) (https://www.ncbi.nlm.nih.gov/) sequence read archive (SRA) with accession number PRJNA576585. After the processing of raw reads through in-house Perl scripts, clean reads were aligned to the soybean genome using HISTAT protocol [30]. Then the reads numbers mapped to each gene were counted using HTSeq software (0.6.1 version) [31] and gene expression levels were determined using the Fragments Per Kilobase of transcript per Million mapped reads (FPKM) [32]. The “ggplots” R-package software was used to show the cluster interactions among samples. A significant false discovery rate-adjusted *P* value (FDR) < 0.05 was used as the empirical parameter to identify the differentially expressed genes (DEGs) as previously described [33]. Furthermore, Gene Ontology (GO) of the DEGs was performed using the “clusterProfiler” R-package [34]. Finally, the Kyoto Encyclopedia of Genes and Genomes (KEGG) enrichment analyses was carried out by Blast software[35].

### Metabolome analysis

Freeze-dried leaf samples were crushed using a mixer mill (MM 400, Retsch) with a zirconia bead for 1.5 min at 30 Hz. Afterward, 100 mg powder was extracted overnight at 4°C with 1.0 mL 70% aqueous methanol. Following centrifugation at 10000 x g for 10 min, the extracts were absorbed (CNWBOND Carbon-GCB SPE Cartridge, 250 mg, 3 mL; ANPEL, Shanghai, China) and filtered (SCAA-104, 0.22 μm pore size; ANPEL, Shanghai, China) before LC-MS analysis. The quantification of metabolites was performed following the multiple reaction monitoring (MRM) analysis procedures using a triple quadrupole-linear ion trap mass spectrometer (QTRAP), API 4500 Q TRAP LC/MS/MS System. Analyses were carried out as described previously [36], with each group having six biological replicates.

### Confirmation of the expression profiles by qRT-PCR

All samples were analyzed using the quantitative real-time PCR (qRT-PCR) technique to validate the transcriptomic results of this study. The same RNA samples for the transcriptome analysis were reverse transcribed using a PrimeScript™ RT reagent Kit with gDNA Eraser (Perfect Real Time) (TaKaRa, Dalian, China) following the manufacturer’s instructions. qRT-PCR procedures were performed on a Real-Time PCR System (CFX96, Bio-Rad, USA) using TB Green® Premix Ex Taq™ II (Tli RNaseH Plus) (TaKaRa Co. Ltd, Dalian, China) according to the manufacturer’s instructions. The primer sequences for specific genes are listed in S2 Table. Relative expression levels of selected genes were calculated using the 2^−ΔΔCt^ method [37], and the soybean gene *GAPDH* served as an internal control.

### Determination of concentrations of quercetin, genistein, glycitein and β-sitosterol

High-performance liquid chromatography (HPLC) was performed to detect the concentrations of quercetin (Catalog Number:A0083, CAS NO:117-39-5), genistein (Catalog Number: A0009, CAS NO: 446-72-0), glycitein (Catalog Number: A0006, CAS NO: 40957-83-3) and β-sitosterol (Catalog Number:A0197, CAS NO:83-46-5). HPLC measurements were carried out using an LC-60 HPLC system (Shimadzu, Japan) consisting of a controller (SPD-20A), a binary pump (LC-20AD), and a column oven (CTO-10ASvp). The separation was achieved with an Xtimate-C18 column (250 mm × 4.6 mm, 5 μm, Japan). 20 mmol/L ammonium acetate and 0.1% formic acid (*v*:*v* = 1:4, pH 4.0) were used as mobile phase to elute. The flow rate was set at 1.0 mL min^-1^, and the injection volume of the sample was 10 μL. Quercetin, genistein, glycitein and β-sitosterol were detected at wavelengths of 360 nm, 260 nm, 260 nm, and 205 nm, respectively.

### Statistical analysis

Data obtained from this research were expressed as the mean ± standard deviation (SD) and analyzed using SPSS 17.0 software (SPSS Inc., Chicago, IL, USA). The statistical significance among the various groups was determined using one-way analysis of variance (ANOVA), followed by Duncan’s multiple range test. Values of *P* < 0.05 were considered to be statistically significant. For metabolome analysis, the relative abundances of each metabolite were converted to log values before analysis to meet normality. Principal components analysis (PCA) on normalized data was performed using the R software (version 3.1.1). Differential metabolites (variable importance in project, VIP ≥ 1) were chosen at fold change ≥ 2 or fold change ≤ 0.5 statistically significant (*P* < 0.05) based on Orthogonality Partial Least Squares-Discriminant Analysis (OPLS-DA).

## Results

### Effect of exogenous melatonin treatment on the drought tolerance

**Fig 1:**
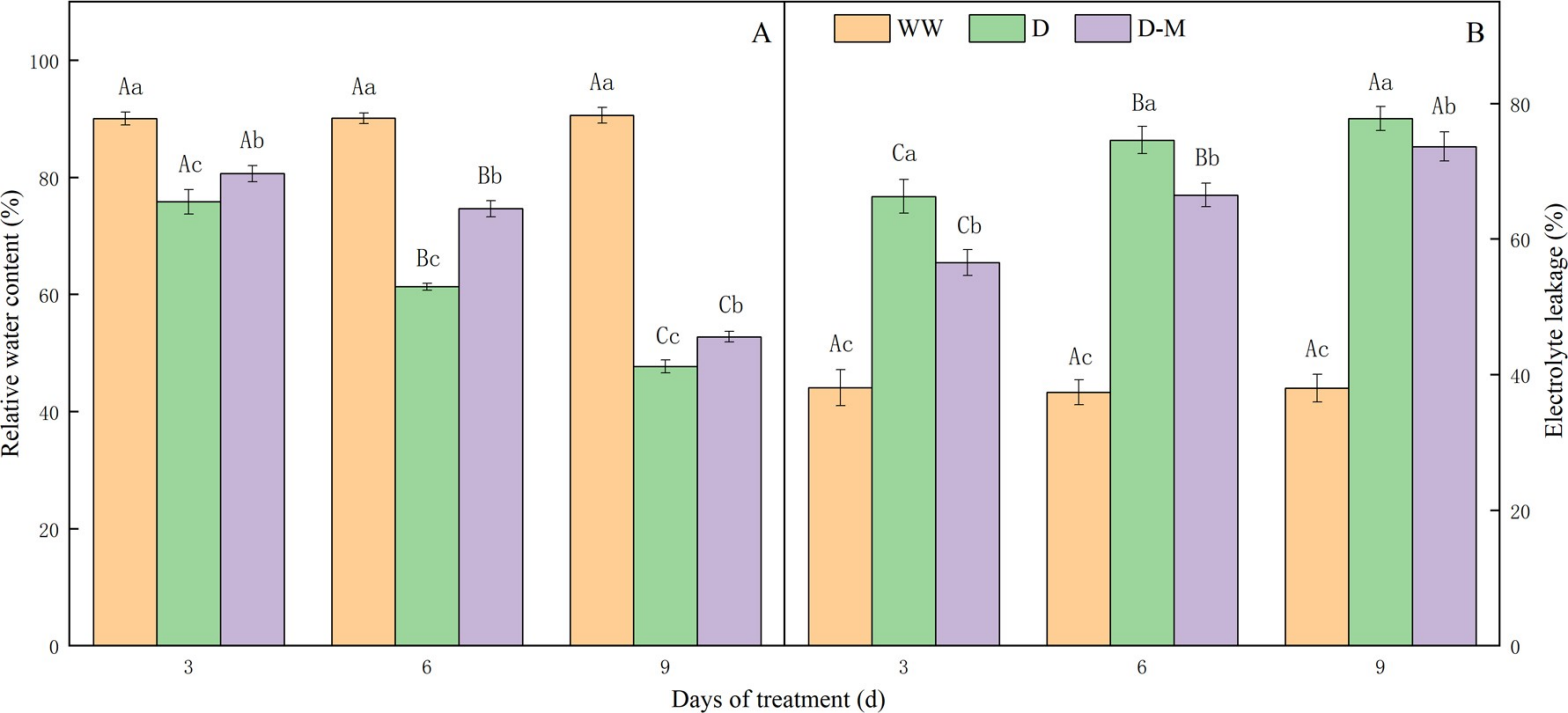
Effect of exogenous melatonin treatment on the relative water content (A) and electrolyte leakage (B) in the leaves of soybean. The values were the means of three replicates of three independent experiments. Different lowercase means there were significant differences (*P* < 0.05) among different treatments of the same day. Different capital letters mean there were significant differences (*P* < 0.05) among different days of the same treatment.

The RWC of soybean leaves on 6 and 9 d in the D or D-M group significantly decreased (*P* < 0.05) when compared to that on 3 d in each group. However, the decrease rate in the D-M group was lower than that in the D group (Fig 1A). The E_L_ of soybean leaves on 6 and 9 d in the D or D-M group significantly increased (*P* < 0.05) when compared to that on 3 d in each group. However, the increase rate in the D-M group was lower than that in the D group (Fig 1B). The RWC of soybean leaves in the D group decreased when compared to the WW group (Fig 1A). After of 3, 6, and 9 d of drought stress, it reduced by 15.83%, 32.22%, and 47.33%, respectively. This trend shows that the RWC of soybean leaves gradually decreased as the drought stress period increased. However, it significantly increased by 6.35%, 21.75% and 10.64% after 3 d, 6 d, and 9 d of exogenous melatonin treatment, respectively (*P* < 0.05). In Fig 1B, E_L_ of soybean leaves in the D group was higher than that in the WW group after of 3, 6, and 9 d of drought stress, it increased by 74.15%, 99.49% and 104.60%, respectively. This observation suggests that there is a positive correlation between drought stress and leaf E_L_. However, it decreased significantly by 14.75%, 10.85% and 5.32% after 3 d, 6 d and 9 d of exogenous melatonin treatment, respectively (*P* < 0.05). Based on the results above, exogenous melatonin treatment could effectively alleviate the decrease of RWC and the increase of E_L_ induced by drought stress.

As shown in Table 1, the pod number per plant, number of grains per plant, 100-seed weight, and grain weight per plant in the D group was significantly lower than that of the WW group (*P* < 0.05), indicating that drought stress could decrease seed yield. Exogenous melatonin treatment did not affect the pod number per plant of soybean under drought stress (P > 0.05) substantially. However, exogenous melatonin treatment significantly increased the number of grains per plant, 100-seed weight and grain weight per plant, respectively (*P* < 0.05), which implied that exogenous melatonin treatment during grain filling stage could effectively prevent the decreases of the number of grains r per plant, 100-seed weight and grain weight per plant to improve the seed yield under drought stress.

**Table 1:** Effects of melatonin on yield of soybean under drought stress.

Treatment	Pod number per plant	Number of grains per plant	100-seed weight/g	grain weight per plant (g)
**WW**	21.30±1.25 ^a^	47.10±0.99 ^a^	23.76±2.78 ^a^	10.83±0.70 ^a^
**D**	19.80±0.79 ^b^	43.30±1.16 ^c^	17.35±0.97 ^c^	7.83±0.60 ^c^
**D-M**	20.20±1.23 ^ab^	45.80±1.03 ^b^	19.59±1.30 ^b^	8.68±0.72 ^b^

Values with different superscript letters were significantly different at *P* < 0.05.

### Transcriptomic characteristics of all samples and differential expression analysis

Transcriptomic tools were used to study drought-induced changes. Here, soybean leaves were collected from the three groups, sequenced using RNA-Seq protocol and subjected to bioinformatics analyses. All treatments showed high correlation between biological replicates (R^2^ > 0.96), thus validating its reliability (S2 Fig). However, correlation within treatment groups was lower. DEGs were identified using two comparisons: WW/D and D/D-M to show the underlying mechanisms by which melatonin confers drought tolerance. As shown in S3 Fig, the WW/D comparison had a total of 5822 DEGs, of which 3508 and 2314 were up-regulated and down-regulated, respectively (S3 Fig). The D/D-M comparison, on the other hand, had only 1132 DEGs, of which 110 and 1022 were up-regulated and down-regulated, respectively.

### DEGs affected by drought stress and exogenous melatonin treatment

The Venn diagram between the WW/D and D/D-M comparisons was constructed to identify DEGs whose expression levels could be reversed by exogenous melatonin treatment (S4A Fig). Drought conditions in the D group significantly induced a total of 5822 genes compared to the WW group. Here, 4970 genes were not affected by melatonin application. On the other hand, 1132 DEGs were identified in the D/D-M comparison, of which 280 genes were not DEGs in the WW/D comparison. There were 852 overlapped DEGs that were both regulated by drought and melatonin in soybean leaves. In addition, the top 50 DEGs were selected by *P*-value. As shown in S4B Fig, most of DEGs were enhanced in the WW/D comparison but were reversed after melatonin treatment, indicating that melatonin treatment could relieve drought stress by regulating these substantial transcriptomic changes.

### Gene enrichment analysis for the DEGs in response to drought

After identifying the DEGs, the GO enrichment analysis was performed. Three categories (biologic process, molecular function and cellular component) comparing the treatment groups (WW/D and D/D-M) are shown in S5 Fig. Most of the DEGs in the WW/D group were detected in the biological process category such as cellular process (2503 genes, approximately 42% of DEGs), metabolic process (2285 genes, about 39% of DEGs), and response to stimulus (1577 genes, approximately 27% of DEGs). In the cellular component class, most of the DEGs were classified into cell (3179 genes, approximately 54% of DEGs), cell part (3176 genes, about 54% of DEGs), and organelle (2199 genes, about 37% of DEGs). In the molecular functions category, most of the DEGs were distributed to binding (2347 genes, about 40% of DEGs), catalytic activity (2219 genes, about 38% of DEGs), and transporter activity (469 genes, about 8% of DEGs).

Most DEGs in the D/D-M comparison were assigned to biological processes such as metabolic process (459 genes, about 40% of DEGs), cellular process (453 genes, about 40% of DEGs), and response to stimulus (345 genes, accounting for about 30% of DEGs). In the cellular component class, the DEGs were assigned to cell (560 genes, about 49% of DEGs), cell part (559 genes, approximately 49% of DEGs), and organelle (357 genes, about 31% of DEGs). In the molecular function category, most of the DEGs were grouped into catalytic activity (510 genes, about 45% of DEGs), binding (453 genes, about 40% of DEGs), and transcription regulator activity (86 genes, about 7% of DEGs). These classification results could help us investigate the role of melatonin in the drought tolerance.

**Fig 2:**
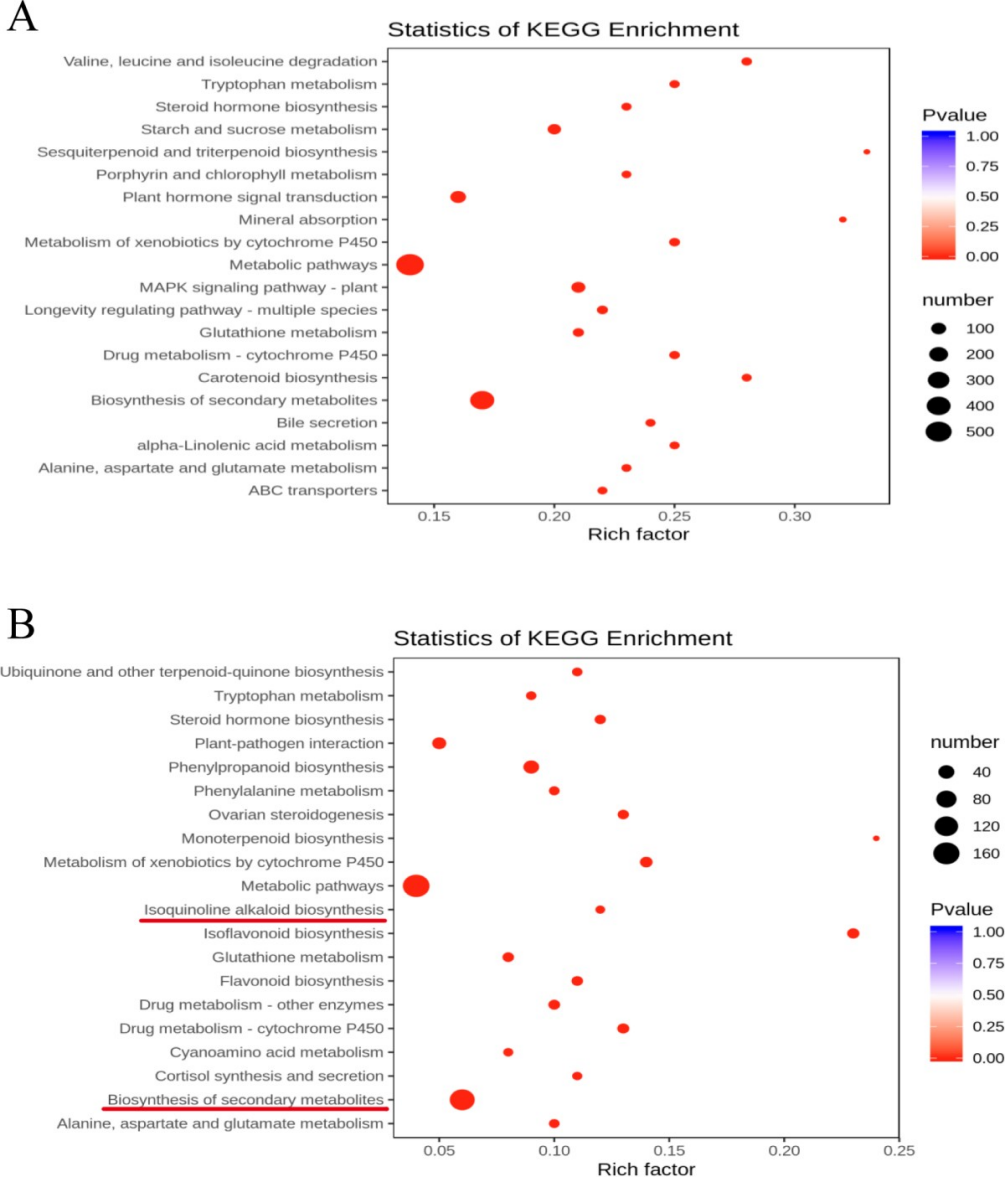
Bubble diagrams of KEGG pathway enrichment for DEGs in (A) WW/D and (B) D/D-M comparison. The rich factor is calculated as the DEGs number divided by the base number of any given pathway. Dot size denotes the number of genes, and dot color denotes the range of adjusted P-value.

Gene enrichment analysis of the DEGs based on the KEGG database are shown in Fig 2. The top 20 enrichments of biological pathways in the WW/D comparison include ‘biosynthesis of secondary metabolites’, ‘MAPK plant signaling pathway’, ‘metabolic pathways’, ‘valine, leucine and isoleucine degradation’, ‘starch and sucrose metabolism’, ‘metabolism of xenobiotics by cytochrome P450’, ‘carotenoid biosynthesis’, ‘drug metabolism-cytochrome P450’, ‘tryptophan metabolism’, ‘longevity regulating pathway-multiple species’, ‘alpha-linolenic acid metabolism’, ‘glutathione metabolism’, ‘bile secretion’, ‘steroid hormone biosynthesis’, ‘alanine, aspartate and glutamate metabolism’, ‘sesquiterpenoid and triterpenoid biosynthesis’, ‘mineral absorption’, ‘MAPK signaling pathway’, ‘plant hormone signal transduction ABC transporters’, and ‘porphyrin and chlorophyll metabolism’ (Fig 2A).

The top 20 enrichments of biological pathways in the D/D-M comparison include ‘biosynthesis of secondary metabolites’, ‘isoflavonoid biosynthesis’, ‘phenylpropanoid biosynthesis’, ‘metabolism of xenobiotics by cytochrome P450’, ‘metabolic pathways’, ‘drug metabolism-cytochrome P450’, ‘ovarian steroidogenesis’, ‘flavonoid biosynthesis’, ‘steroid hormone biosynthesis’, ‘drug metabolism-other enzymes’, ‘alanine, aspartate and glutamate metabolism’, ‘ubiquinone and other terpenoid-quinone biosynthesis’, ‘phenylalanine metabolism’, ‘glutathione metabolism’, ‘cortisol synthesis and secretion’, ‘isoquinoline alkaloid biosynthesis’, ‘tryptophan metabolism’, ‘monoterpenoid biosynthesis’, ‘plant-pathogen interaction’ and ‘cyanoamino acid metabolism’ (Fig 2B). The genes that differentially expressed in the D/D-M comparison but not in the WW/D comparison were enriched in these metabolic pathways, besides ‘biosynthesis of secondary metabolites’ pathway, indicating that they may play important roles in the rapid adaptive response to drought stress by melatonin in soybean. Interestingly, ‘biosynthesis of secondary metabolites’ was common to both groups, suggesting that exogenous melatonin treatment could partially treat these changes triggered by drought stress. These pathways were then further analyzed in this report.

### Metabolic analysis of soybean in response to drought

Metabolites were detected in soybean leaves using metabolome techniques to study drought-induced changes. PCA analysis results show that all the groups were distinct but the distance between the WW and D-M groups were closer than between the WW and D groups (S6 Fig). A total of 706 compounds were detected, including amino acids, lipids, organic acids, sugars, alkaloids, amines, flavonoids, and terpenoids. In the D group, a total of 204 differentially-accumulated metabolites (108 up-regulated metabolites and 96 down-regulated metabolites) were found when compared to the WW group (S7A Fig). In the D/D-M comparison, 115 differentially accumulated metabolites (35 up-regulated metabolites and 80 down-regulated metabolites) were identified. Among these differentially accumulated metabolites, 141 differentially accumulated metabolites in the WW/D comparison were not affected by melatonin treatment, and 52 differentially-accumulated metabolites in the D/D-M comparison were not presented in the WW/D comparison. In addition, 63 overlapping differentially accumulated metabolites were both regulated by drought and melatonin in soybean leaf samples.

The 10 most up-regulated and down-regulated metabolites in the WW/D and D/D-M comparisons are shown in S7B and S7C Figs, respectively. There were enhanced levels of 2-phenyl ethanol, benzoic acid, angelicin, 2-picolinic acid, eriodictyol, 1-aminocyclopropanecarboxylic acid, lysine butyrate, lysoPC 16:2, D(+)-melezitose-O-rhamnoside and lysoPC 15:1 were enhanced in the WW/D comparison. However, chalcone, selgin O-hexosyl-O-hexoside, tricin 5-O-hexosyl-O-hexoside, 6-hydroxynicotinic acid, biotin, tricin 7-O-hexosyl-O-hexoside, methyl quercetin O-hexoside, tricin 5-O-hexoside, malvidin-3,5-diglucoside, and petunidin-3,5-diglucoside levels were decreased. Up-regulated metabolites include fumaric acid, selgin O-hexosyl-O-hexoside, biotin, S-(methyl)-glutathione, chrysoeriol O-sinapoylhexoside, 4-guanidinobutyric acid, catechol, herbacetin, 6-hydroxymelatonin, 1,5-diaminopentane. In contrast, down-regulated metabolites were lysoPE 18:1, formononetin (4'-O-methyldaidzein), eriodictyol, nicotinic acid adenine dinucleotide, lysoPC 15:1, lysoPC 16:2, (-)-epiafzelechin, 2-picolinic acid, melatonin, lysoPE 18:2 in the D/D-M comparison. Notably, eriodictyol (flavanone), 2-picolinic acid (organic acids and derivatives), and lysoPC 15:1 (lipids) were up-regulated in the WW/D comparison, but were suppressed by melatonin treatment. Also, selgin O-hexosyl-O-hexoside (flavone) and biotin (vitamins and derivatives) were inhibited in the WW/D comparison, while were promoted in the D/D-M comparison.

### KEGG enrichment analysis

**Fig 3:**
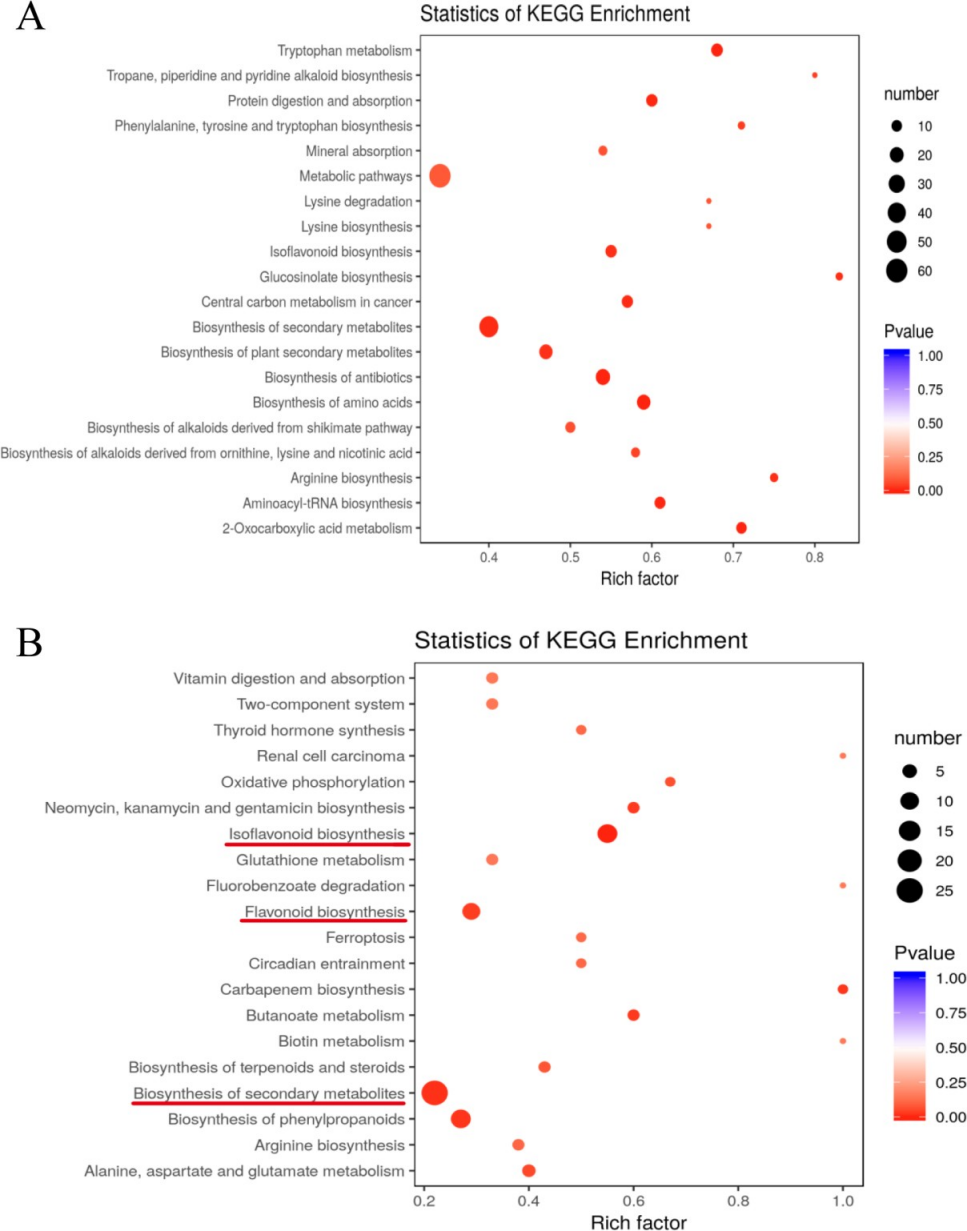
Bubble diagrams of KEGG pathway enrichment for differentially-accumulated metabolites in (A) WW/D and (B) D/D-M comparison. The rich factor is calculated as the number of differentially-accumulated metabolites divided by the base number of any given pathway. Dot size denotes the number of differentially-accumulated metabolites, and dot color denotes the range of adjusted P-value.

Metabolites affected by drought stress were further studied using metabolic mapping analysis. KEGG analysis results show that 113 and 77 pathways were assigned to the WW/D and D/D-M comparisons, respectively. The top 20 enriched biological pathways in the WW/D comparison include ‘biosynthesis of amino acids’, ‘tryptophan metabolism’, ‘biosynthesis of antibiotics’, ‘2-oxocarboxylic acid metabolism’, ‘protein digestion and absorption’, ‘aminoacyl-tRNA biosynthesis’, ‘biosynthesis of secondary metabolites’, ‘central carbon metabolism in cancer’, ‘glucosinolate biosynthesis’, ‘arginine biosynthesis’, ‘isoflavonoid biosynthesis’, ‘biosynthesis of plant secondary metabolites’, ‘phenylalanine, tyrosine and tryptophan biosynthesis’, ‘tropane, piperidine and pyridine alkaloid biosynthesis’, ‘biosynthesis of alkaloids derived from ornithine, lysine and nicotinic acid’, ‘biosynthesis of alkaloids derived from shikimate pathway’, ‘mineral absorption’, ‘lysine degradation’, ‘lysine biosynthesis’, and ‘metabolic pathways’ (Fig 3A). These findings suggest that these metabolic pathways may play crucial roles in drought stress in soybean. In Fig 3B, the 20 most enriched biological pathways in the D/D-M comparison include ‘isoflavonoid biosynthesis’, ‘biosynthesis of secondary metabolites’, ‘biosynthesis of phenylpropanoids’, ‘carbapenem biosynthesis’, ‘butanoate metabolism’, ‘neomycin, kanamycin and gentamicin biosynthesis’, ‘flavonoid biosynthesis’, ‘oxidative phosphorylation’, ‘biosynthesis of terpenoids and steroids’, ‘arginine biosynthesis’, ‘ferroptosis’, ‘thyroid hormone synthesis’, ‘circadian entrainment’, ‘vitamin digestion and absorption’, ‘glutathione metabolism’, ‘two-component system’, ‘fluorobenzoate degradation’, ‘biotin metabolism’, ‘renal cell carcinoma’ and ‘alanine, aspartate and glutamate metabolism’. The metabolites that differentially accumulated in the D/D-M comparison but not in the WW/D comparison were enriched in these metabolic pathways, except for ‘biosynthesis of secondary metabolites’ pathway, indicating that they may play important roles in the rapid adaptive response to drought stress by melatonin in soybean. In addtion, it was observed the ‘biosynthesis of secondary metabolites’ pathway were both regulated by drought stress and exogenous melatonin treatment. It may suggest that melatonin treatment could partially reverse the 'biosynthesis of secondary metabolites' pathway, which was induced by drought.

### Association of transcriptomic and metabolomic changes involved in crucial biological pathways

In this section, we focused on the connection between drought-responsive gene expression and metabolite changes to gain more understanding of the physiological changes in tolerance to drought stress in soybean. Most genes encoding key enzymes and crucial metabolites had positive correlation (quadrant 3 and 7), other genes encoding some essential proteins and metabolites had negative correlation (quadrant 1, 2, 4, 6, 8 and 9), implying that these enzyme-coding genes may play critical roles in the formation or breakdown of important metabolites that lower drought stress (S8 Fig). Further studies are warranted to validate this hypothesis.

### Effect of melatonin on the biosynthesis of secondary metabolites in soybean during drought stress

**Fig 4:**
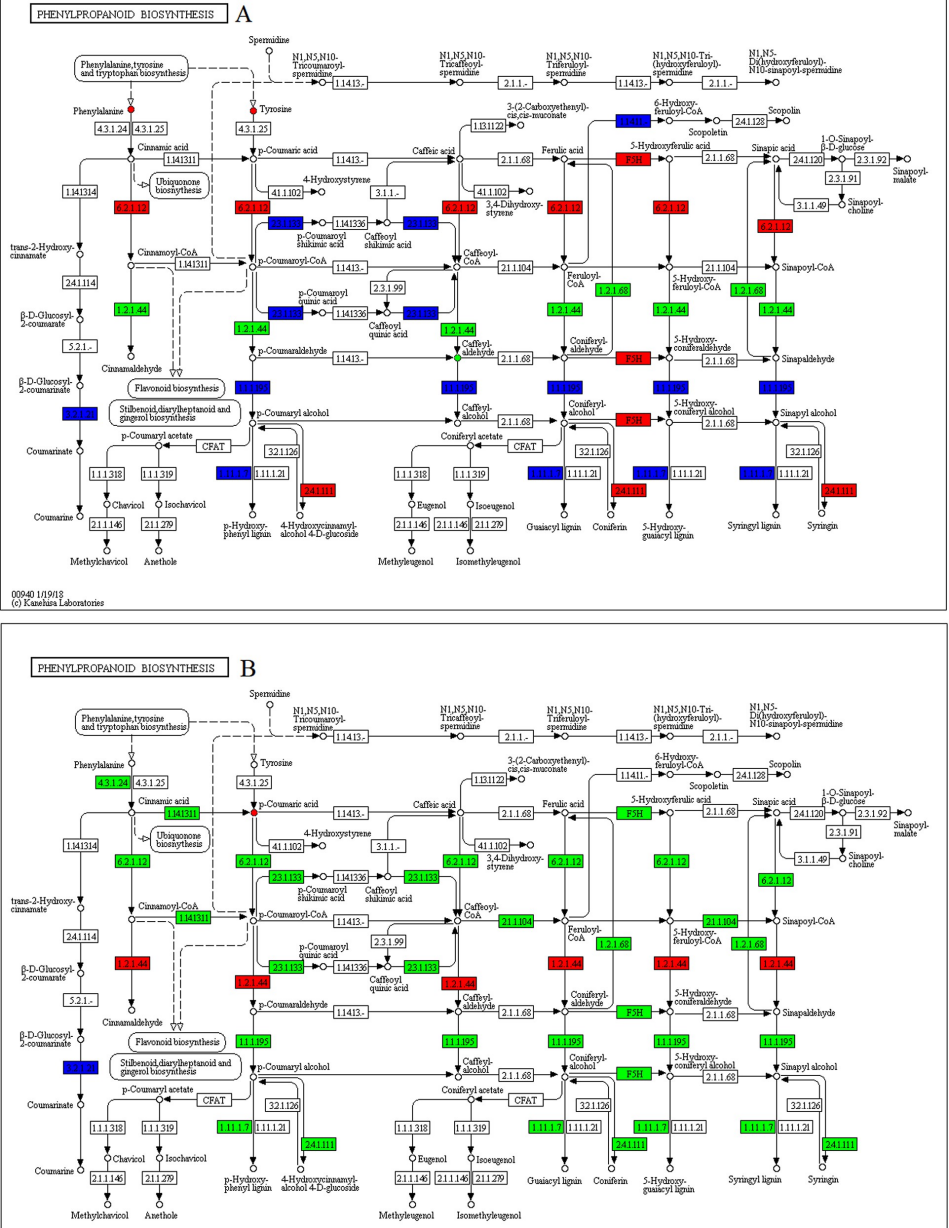
Illustration of KEGG pathway of phenylpropanoid biosynthesis in (A) WW/D comparison and (B) D/D-M comparison. Red means up-regulated genes and metabolites, green means down-regulated genes and metabolites, blue means composition of up-regulated and down-regulated genes.

The expressions of many structural enzyme-coding genes of the phenylpropanoid pathway were significantly affected by drought stress (Fig 4). These genes were, however, not involved in phenolic acid biosynthesis. Therefore, there were no differentially accumulated metabolites related to phenolic acids such as, p-coumaric, ferulic, and caffeic acids. Exogenous application of melatonin decreased p-coumaric accumulation, but increased the expression of the two genes encoded for phenylalanine ammonia-lyase [EC:4.3.1.24] and a gene annotated as trans-cinnamate 4-monooxygenase [EC:1.14.14.91].

**Fig 5:**
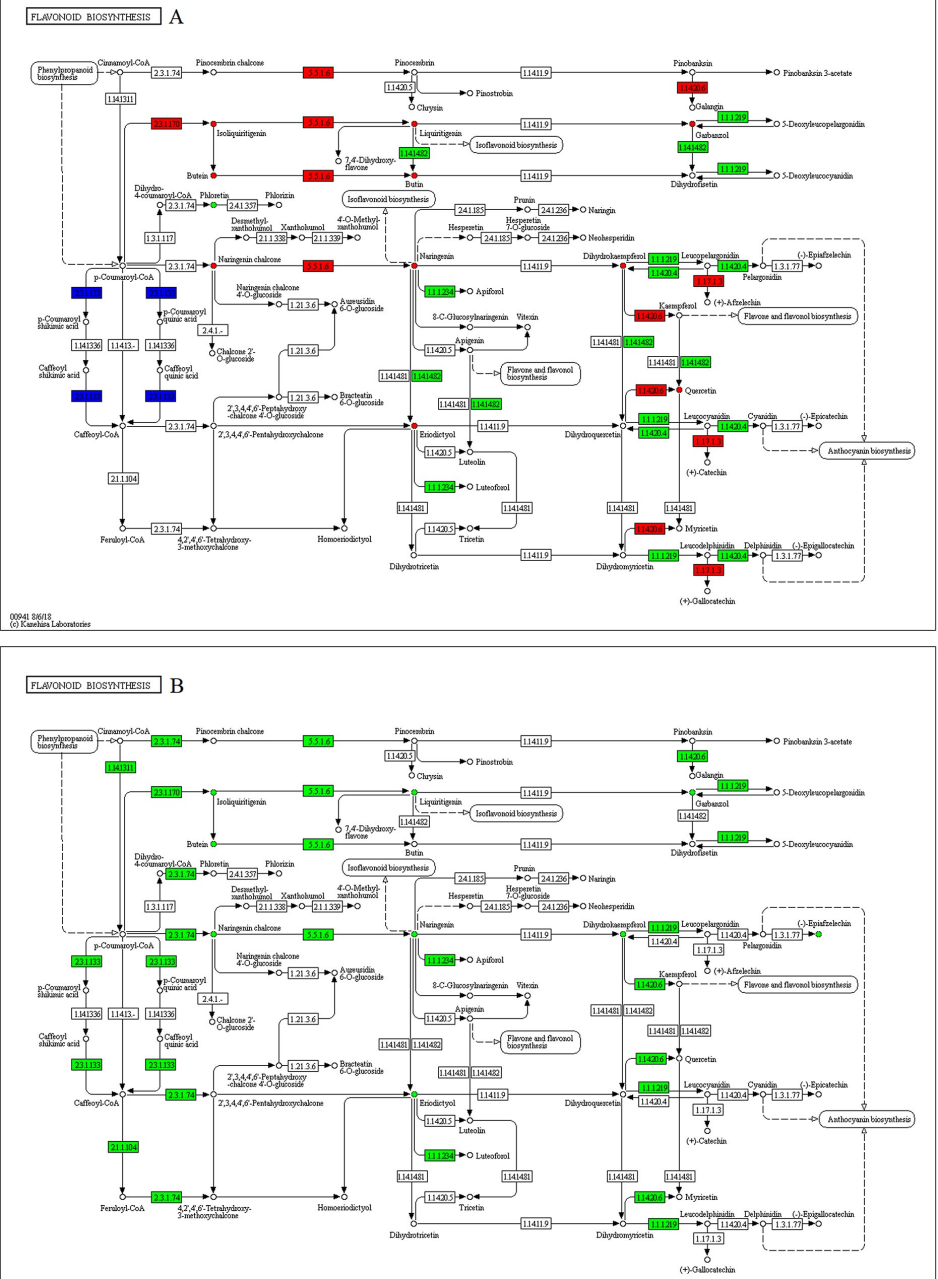
Illustration of KEGG pathway of flavonoid biosynthesis in (A) WW/D comparison and (B) D/D-M comparison. Red means up-regulated genes and metabolites, green means down-regulated genes and metabolites, blue means composition of up-regulated and down-regulated genes.

The detailed expression patterns of the genes involved in flavonoid biosynthesis pathways and their metabolites are presented in Fig 5. Compared with the WW group, the concentrations of isoliquiritigenin, butein, butin, biquiritigenin, garbanzol, naringenin chalcone, naringenin, eriodictyol, dihydrokaempferol and quercetin were lowered in the D group. Interestingly, these flavonoids’ levels were elevated after exogenous melatonin treatment. In addition, the expression of most structural flavonoid genes, such as chalcone synthase [EC:2.3.1.74], trans-cinnamate 4-monooxygenase [EC:1.14.14.91], chalcone isomerase [EC:5.5.1.6], shikimate O-hydroxycinnamoyltransferase [EC:2.3.1.133], caffeoyl-CoA O-methyltransferase [EC:2.1.1.104], bifunctional dihydroflavonol 4-reductase/flavanone 4-reductase [EC:1.1.1.219 1.1.1.234] and flavonol synthase [EC:1.14.20.6] were increased.

**Fig 6:**
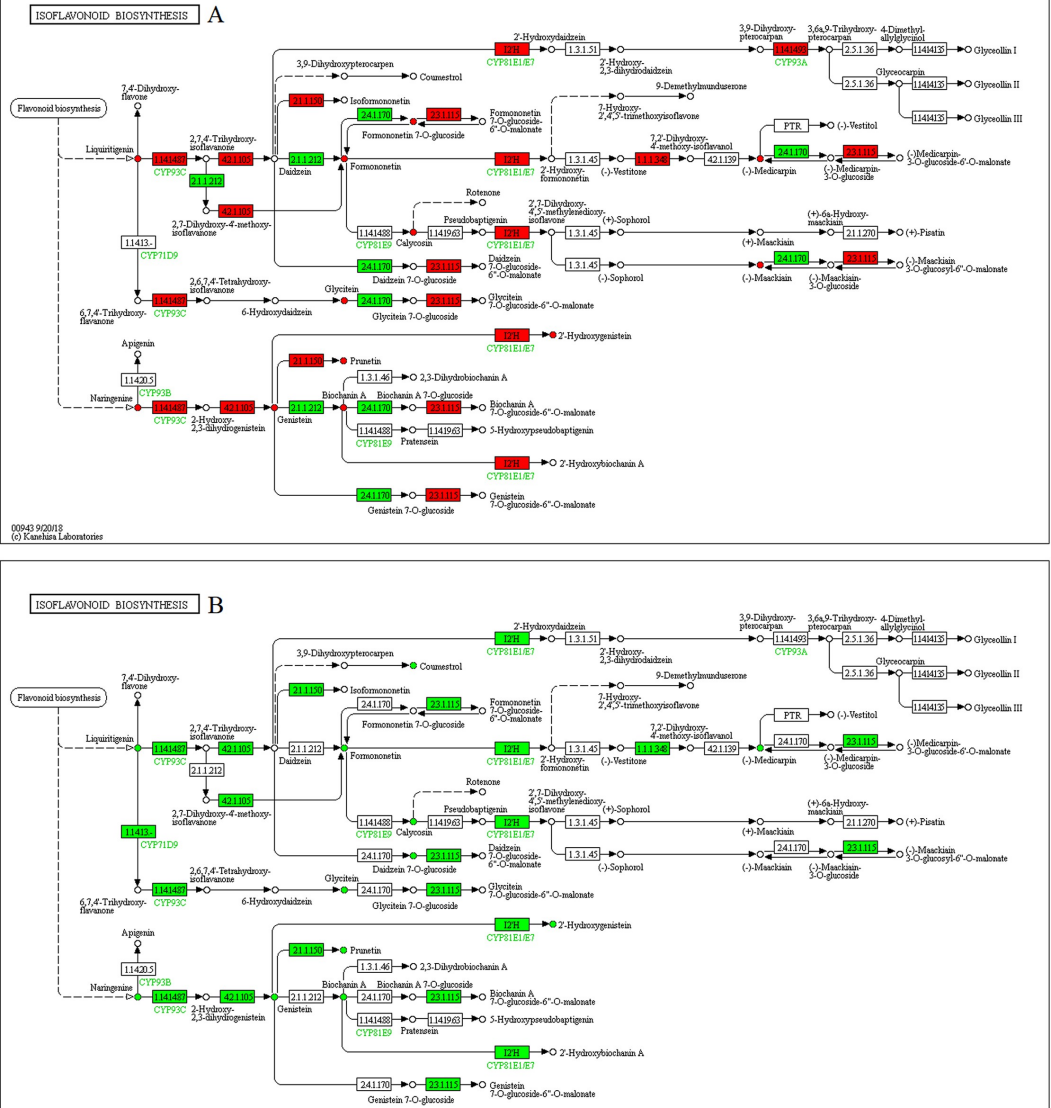
Illustration of KEGG pathway of isoflavonoid biosynthesis in (A) WW/D comparison and (B) D/D-M comparison. Red means up-regulated genes and metabolites, green means down-regulated genes and metabolites, blue means composition of up-regulated and down-regulated genes.

Isoflavonoid biosynthesis was also affected by drought stress and exogenous melatonin treatment (Fig 6). Most of DEGs were down-regulated under drought stress except two DEGs encoded for 2,7,4'-trihydroxyisoflavanone 4'-O-methyltransferase [EC:2.1.1.212] and isoflavone 7-O-glucosyltransferase [EC:2.4.1.170]. Correspondingly, drought stress inhibited the conversion of flavonoid into isoflavonoid such as formononetin (4'-O-methyldaidzein), orobol (5,7,3',4'-tetrahydroxyisoflavone), genistein (4',5,7-trihydroxyisoflavone), calycosin, biochanin A, glycitein, 2'-hydroxygenistein, prunetin, formononetin 7-O-glucoside (ononin). Again, melatonin administration facilitated the modification of flavonoid into the aforementioned isoflavonoids with a corresponding up-regulation of all DEGs associated with isoflavonoid biosynthesis.

**Fig 7:**
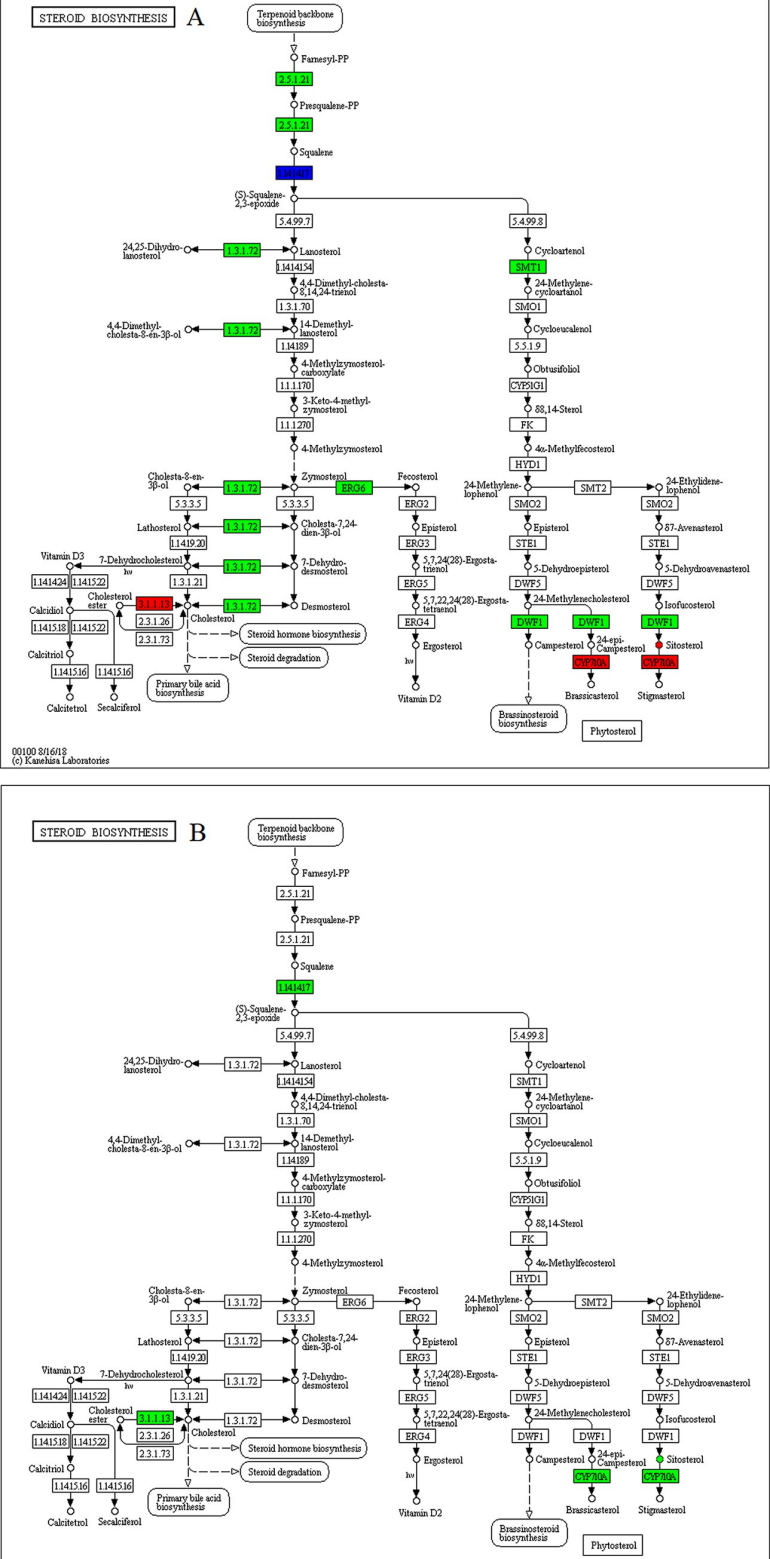
Illustration of KEGG pathway of steroid biosynthesis in (A) WW/D comparison and (B) D/D-M comparison. Red means up-regulated genes and metabolites, green means down-regulated genes and metabolites, blue means composition of up-regulated and down-regulated genes.

In the steroid biosynthesis pathway (Fig 7), among the DEGs, genes related to β-sitosterol biosynthesis, such as farnesyl-diphosphate farnesyltransferase [EC:2.5.1.21], sterol 24-C-methyltransferase [EC:2.1.1.41] and Delta(24)-sterol reductase [EC:1.3.1.72 1.3.1.-] were upregulated under drought stress, but the D group have a lower content of β-sitosterol than that of the WW group. Exposure to melatonin treatments significantly increased the density of β-sitosterol.

### Validation of uniqueness expression using qRT-PCR

**Fig 8:**
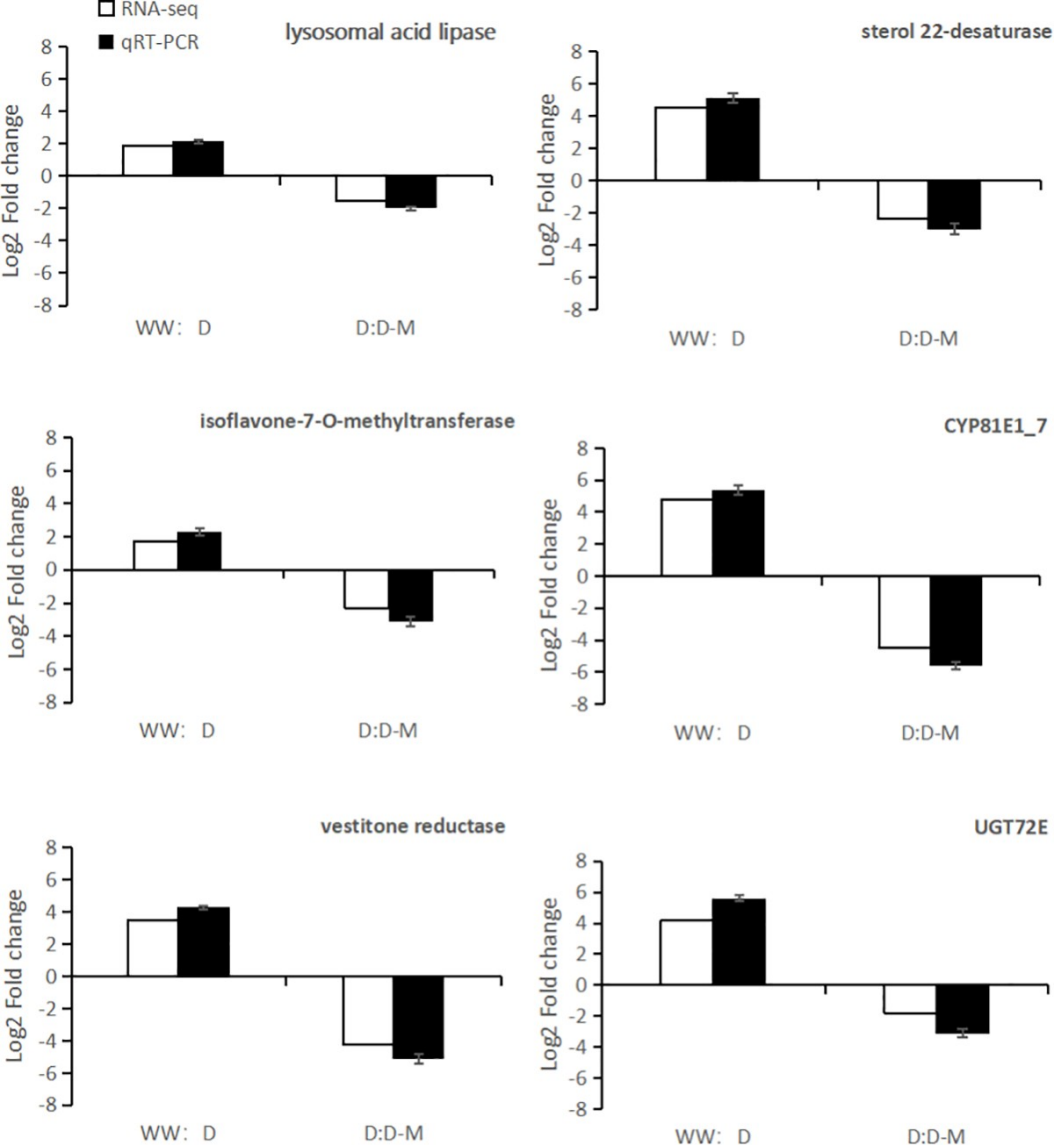
Validation of transcriptomic results by qRT-PCR. The white bar signifies transcriptomic data for the DEGs, and the black bar denotes qRT-PCR results for the DEGs. The values were the means of three replicates of three different experiments.

The transcriptomic analysis results of this study were further validated using the qRT-PCR technique. Here, 6 DEGs, including lysosomal acid lipase, sterol 22-desaturase, isoflavone-7-O-methyltransferase, CYP81E1_7, vestitone reductase and UGT72E, were selected. The expression patterns of these DEGs were in consonance with the earlier observed transcriptomic data (Fig 8).

### Confirm the metabolomic profile using HPLC

**Fig 9:**
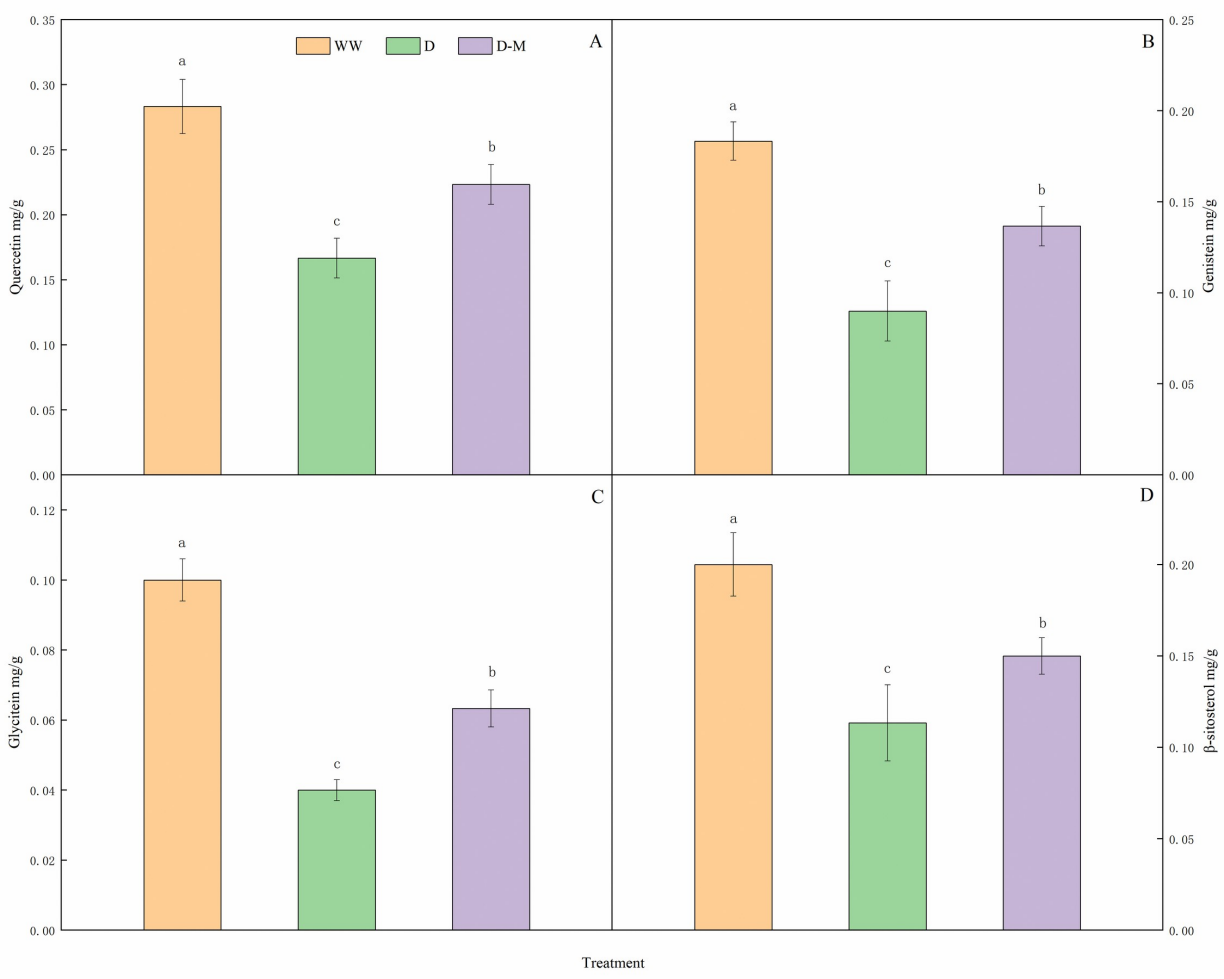
Concentrations of quercetin (A), genistein (B), glycitein (C) and β-sitosterol (D) in the leaves of soybean. The values were the means of three replicates of three independent experiments. Values with different superscript letters were significantly different at *P* < 0.05.

The metabolomic changes were further confirmed using the HPLC technique. Here, 4 differentially accumulated metabolites, including quercetin, genistein, glycitein and β-sitosterol were selected, because they were differentially accumulated metabolites in the flavonoid biosynthesis, isoflavonoid biosynthesis and steroid biosynthesis pathways from KEGG analysis, which play vital roles for improvement of drought tolerance in soybean by exogenous melatonin treatment. The representative chromatograms of HPLC were shown in S9–S11 Figs. The concentrations of quercetin, genistein, glycitein and β-sitosterol in the D group were lower than those in the WW group (Fig 9). However, melatonin treatment significantly increased their concentrations in the leaves when compared with the D group (*P* < 0.05). These results were consistent with the metabolomics profile earlier reported that quercetin, genistein, glycitein and β-sitosterol in the WW/D comparison were up-regulated metabolites, while were down-regulated metabolites in the D/D-M comparison.

## Discussions

As an important world legume, soybean (*Glycine max* L.) is a major source of oil and protein for millions of people and livestock. However, drought stress triggered by climate change factors can result in significant losses as well as the reduction of seed quality [38]. Therefore, it is necessary to explore effective measures to alleviate the effects of drought stress on soybean. In 1995, melatonin, a plant growth regulator (PGR), was isolated from higher plants and functions in growth stimulation and boosting plant resistance [39]. Notably, the negative effects of drought stress were reversed by exogenous melatonin treatment in some plants, including apple [40], grape [41], cucumber [42], tomato [43], wheat [21] and maize [44]. In our previous study, it was demonstrated that exogenous melatonin treatment could improve the tolerance of drought-treated soybean [24]. However, the specific mechanisms by which melatonin alleviates drought stress is still unclear. In the present study, we reported the role of melatonin in resistance to drought stress using transcriptomic and metabolomic analysis, which could identify candidate genes and key metabolic pathways involved in drought response in soybean. Our results showed that the exogenous melatonin treatment could increase the relative water content and decrease the electrolyte leakage in leaves and increase seed yield under drought stress.

Data analyses using transcriptomic and metabolomic techniques can give more information into complex interactions between and within several factors. Thus, we analyzed how melatonin response to drought stress by integrating transcriptomic and metabolomic approaches. A combination of transcriptomic and metabolomic analysis could highlight the vital role of exogenous melatonin for the improvement of drought tolerance in soybean. The KEGG enrichment analysis was carried out to show the biological pathways of the DEGs and differentially-accumulated metabolites. It is noteworthy that pathways involved in ‘biosynthesis of secondary metabolites’ including phenylpropanoid, flavonoid, isoflavonoid, and steroid biosynthesis pathways were significantly enriched in both the WW/D and D/D-M comparisons through transcriptome and metabolome. Secondary metabolites abound in different levels in different plants, and their presence is most noticeable under stress conditions. External application of phenolic compounds can confer some drought tolerance characteristics, although these vary from one plant species to another [45]. In this study, two genes encoding phenylalanine ammonia-lyase (PAL) expression were down-regulated under drought stress, but increased after exogenous melatonin treatment. PAL expression has been linked to drought stress responses previously. In addition, PAL can convert phenylalanine to fuel the phenylpropanoid metabolism pathway [46]. However, drought stress did not change the phenolic acid contents of samples. At the level of phenolic acids under abiotic stresses, previous data indicated some possible reasons. Bettaieb et al. demonstrated that the production of phenolic acids could be in response to the level of water deficit [47], and this may explain why there was no change in phenolic acid levels under drought stress as observed in this study.

Aside from playing integral roles in plant development and reproduction, flavonoids are also important for plant stress defense [48]. This feature has been shown previously under induced stress conditions in *Ligustrum vulgare* [49], *Scutellaria baicalensis* [50], and rice [51]. Our study showed that melatonin treatment improved flavonoids accumulation. Flavinoids may also shield plants from conditions imposed by water deficiency [52]. Naghizadeh et al. recently reported that exogenous melatonin application mitigates the adverse effects of drought stress on morpho-physiological traits and secondary metabolites including flavonoid in Moldavian balm (*Dracocephalum moldavica*) [53]. Furthermore, Liang et al. reported that exogenous melatonin administration could delay the senescence of Kiwifruit Leaves by regulating the antioxidant capacity and biosynthesis of flavonoids [54]. The possible dual roles of flavinoids have also been posited, suggesting that these compounds maintain high antioxidant properties by blocking the production of reactive oxygen species (ROS) [55] and acting as ROS scavengers once ROS are produced [52]. These findings indicated that melatonin might have a role in the promotion of the antioxidant capacity of soybeans through secondary metabolism regulation.

Many plant species contain an active phytosterol called β-sitosterol, which functions in maintaining the lipid bilayers membrane integrity. Previous studies have also implicated this compound in conferring plants with stress tolerance features. Recently, the treatment of tomato seeds with β-sitosterol conferred heightened resistance during extreme temperature conditions, implying that this phytosterol can have drought-resistant characteristics [56]. Its external application significantly lowered stress caused by high salt levels in pepper (*Capsicum annum*) and sunflower (*Helianthus annuus*), as shown by growth and physiological analyses [57, 58]. Metabolomics and physiological analyses revealed that β‑sitosterol could improve white clover growth processes and resistance to water deficits [59]. In the steroid biosynthesis pathway, exposure to melatonin treatment significantly increased the density of β-sitosterol, indicating that melatonin provided beneficial effects in alleviating drought stress by regulating β-sitosterol production.

## Conclusion

In summary, findings from this study showed that exogenous melatonin treatment could increase the relative water content and decrease the electrolyte leakage in leaves and increase seed yield under drought stress. Key metabolic pathways and metabolic products induced by exogenous melatonin treatment under drought conditions were also reported. Exogenous melatonin application could mitigate the adverse effects of drought stress by modulating ‘biosynthesis of secondary metabolites’ pathways including phenylpropanoid, flavonoid, isoflavonoid, and steroid biosynthesis, which regulate soybean leaf response to water deficit. Furthermore, this study gave insights into the molecular mechanisms regulating drought stress reduction by melatonin application in soybean. It also provides useful evidence for developing melatonin-based agents to protect soybean crops from drought conditions.

## Supporting information

S1 Table The growth temperature. (DOCX)

S2 Table Primer sequences of DEGs for RT-qPCR. (DOCX)

S1 Fig The photo of potted plants during grain filling stage. (DOCX)

S2 Fig Pearson correlation coefficients from all genes between each pair of samples of transcriptome. (DOCX)

S3 Fig Volcano plots of DEGs in (A) WW/D and (B) D/D-M comparisons. Black dots represent genes without significant differential expression; red and green dots denote significantly up-regulated and down-regulated genes respectively in the WW/D and D/D-M comparisons.
(DOCX)

S4 Fig Drought stress and exogenous melatonin treatment affect alterations in transcriptome. (A) The Venn diagram shows the overlapped DEGs between the WW/D and D/D-M comparisons. (B) Heat maps of the overlaped DEGs between the WW/D and D/D-M comparisons.
(DOCX)

S5 Fig Gene Ontology (GO) classifications of differentially expressed genes (DEGs). (A) GO analysis of DEGs in the WW/D comparison; and (B) GO analysis of DEGs in the D/D-M comparison.
(DOCX)

S6 Fig PCA analysis of metabonomics. (DOCX)

S7 Fig Drought stress and exogenous melatonin treatment affect alterations in metabolome. (A) The Venn diagram shows the overlapped differentially-accumulated metabolites between the WW/D and D/D-M comparisons, (B) Histogram of the differentially-accumulated metabolites in the WW/D comparison, and (C) Histogram of the differentially-accumulated metabolites in the D/D-M comparison.
(DOCX)

S8 Fig Conjoint analysis between transcriptomic and metabolomic changes by nine-quadrants. (A) WW/D comparison and (B) D/D-M comparison.
(DOCX)

S9 Fig The representative HPLC chromatograms of quercetin detecting at wavelength of 360 nm. (A) HPLC chromatogram of quercetin in standard solution; (B) HPLC chromatogram of quercetin in the leaf of soybean in the WW group; (C) HPLC chromatogram of quercetin in the leaf of soybean in the D group; and (D) HPLC chromatogram of quercetin in the leaf of soybean in the D-M group.
(DOCX)

S10 Fig The representative HPLC chromatograms of genistein and glycitein detecting at wavelength of 260 nm. (A) HPLC chromatogram of genistein and glycitein in standard solution; (B) HPLC chromatogram of genistein and glycitein in the leaf of soybean in the WW group; (C) HPLC chromatogram of genistein and glycitein in the leaf of soybean in the D group; and (D) HPLC chromatogram of genistein and glycitein in the leaf of soybean in the D-M group.
(DOCX)

S11 Fig The representative HPLC chromatograms of β-sitosterol detecting at wavelength of 205 nm. (A) HPLC chromatogram of β-sitosterol in standard solution; (B) HPLC chromatogram of β-sitosterol in the leaf of soybean in the WW group; (C) HPLC chromatogram of β-sitosterol in the leaf of soybean in the D group; and (D) HPLC chromatogram of β-sitosterol in the leaf of soybean in the D-M group.
(DOCX)

## References

[1] Hon-Ming L, Xun X, Xin L, Wenbin C, Guohua Y, et al (2010) Resequencing of 31 wild and cultivated soybean genomes identifies patterns of genetic diversity and selection. Nature Genetics 42: 1053–1059. 10.1038/ng.715 21076406

[2] Le DT, Nishiyama R, Watanabe Y, Tanaka M, Seki M, et al (2012) Differential gene expression in soybean leaf tissues at late developmental stages under drought stress revealed by genome-wide transcriptome analysis. PloS one 7: e49522 10.1371/journal.pone.0049522 23189148 PMC3505142

[3] Akpınar BA, Lucas SJ, Budak H (2013) Genomics approaches for crop improvement against abiotic stress. The Scientific World Journal 2013:361921 10.1155/2013/361921 23844392 PMC3690750

[4] Xu C, Xia C, Xia Z, Zhou X, Huang J, et al (2018) Physiological and transcriptomic responses of reproductive stage soybean to drought stress. Plant cell reports 37: 1611–1624. 10.1007/s00299-018-2332-3 30099610

[5] Tran L-SP, Urao T, Qin F, Maruyama K, Kakimoto T, et al (2007) Functional analysis of AHK1/ATHK1 and cytokinin receptor histidine kinases in response to abscisic acid, drought, and salt stress in Arabidopsis. Proceedings of the National Academy of Sciences 104: 20623–20628. 10.1073/pnas.0706547105 PMC2154481 18077346

[6] Yamaguchi-Shinozaki K, Shinozaki K (2006) Transcriptional regulatory networks in cellular responses and tolerance to dehydration and cold stresses. Annu Rev Plant Biol 57: 781–803. 10.1146/annurev.arplant.57.032905.105444 16669782

[7] Valliyodan B, Nguyen HT (2006) Understanding regulatory networks and engineering for enhanced drought tolerance in plants. Current opinion in plant biology 9: 189–195. 10.1016/j.pbi.2006.01.019 16483835

[8] Yang S, Vanderbeld B, Wan J, Huang Y (2010) Narrowing down the targets: towards successful genetic engineering of drought-tolerant crops. Molecular plant 3: 469–490. 10.1093/mp/ssq016 20507936

[9] Ma Y, Qin F, Tran L-SP (2012) Contribution of genomics to gene discovery in plant abiotic stress responses. Molecular plant 5: 1176–1178. 10.1093/mp/sss085 22930735

[10] Tan D-X, Hardeland R, Manchester LC, Korkmaz A, Ma S, et al (2011) Functional roles of melatonin in plants, and perspectives in nutritional and agricultural science. Journal of experimental botany 63: 577–597. 10.1093/jxb/err256 22016420

[11] Hardeland R (2019) Melatonin in the evolution of plants and other phototrophs. Melatonin Research 2: 10–36.

[12] Posmyk MM, Kuran H, Marciniak K, Janas KM (2008) Presowing seed treatment with melatonin protects red cabbage seedlings against toxic copper ion concentrations. Journal of Pineal Research 45: 24–31. 10.1111/j.1600-079X.2007.00552.x 18205729

[13] Szafrańska K, Glińska S, Janas K (2013) Ameliorative effect of melatonin on meristematic cells of chilled and re-warmed Vigna radiata roots. Biologia plantarum 57: 91–96.

[14] Yin L, Wang P, Li M, Ke X, Li C, et al (2013) Exogenous melatonin improves M alus resistance to M arssonina apple blotch. Journal of Pineal Research 54: 426–434. 23356947 10.1111/jpi.12038

[15] Li C, Zhao Q, Gao T, Wang H, Zhang Z, et al (2018) The mitigation effects of exogenous melatonin on replant disease in apple. Journal of pineal research 65: e12523 10.1111/jpi.12523 30230015

[16] Wei Z, Li C, Gao T, Zhang Z, Liang B, et al (2019) Melatonin increases the performance of Malus hupehensis after UV-B exposure. Plant Physiology and Biochemistry 139: 630–641. 10.1016/j.plaphy.2019.04.026 31039504

[17] Wang P, Sun X, Li C, Wei Z, Liang D, et al (2013) Long‐term exogenous application of melatonin delays drought‐induced leaf senescence in apple. Journal of Pineal Research 54: 292–302. 23106234 10.1111/jpi.12017

[18] Liu J, Wang W, Wang L, Yan S (2015) Exogenous melatonin improves seedling health index and drought tolerance in tomato. Plant Growth Regulation 77: 317–326.

[19] Antoniou C, Chatzimichail G, Xenofontos R, Pavlou JJ, Panagiotou E, et al (2017) Melatonin systemically ameliorates drought stress‐induced damage in M edicago sativa plants by modulating nitro‐oxidative homeostasis and proline metabolism. Journal of Pineal Research 62: e12401. 10.1111/jpi.12401 28226194

[20] Huang B, Chen Y-E, Zhao Y-Q, Ding C-B, Liao J-Q, et al (2019) Exogenous melatonin alleviates oxidative damages and protects photosystem II in maize seedlings under drought stress. Frontiers in plant science 10:677 10.3389/fpls.2019.00677 31178885 PMC6543012

[21] Cui G, Zhao X, Liu S, Sun F, Zhang C, et al (2017) Beneficial effects of melatonin in overcoming drought stress in wheat seedlings. Plant Physiology and Biochemistry 118: 138–149. 10.1016/j.plaphy.2017.06.014 28633086

[22] Campos CN, Ávila RG, de Souza KRD, Azevedo LM, Alves JD (2019) Melatonin reduces oxidative stress and promotes drought tolerance in young *Coffea arabica* L. plants. Agricultural water management 211: 37–47.

[23] Sharma Zheng (2019) Melatonin Mediated Regulation of Drought Stress: Physiological and Molecular Aspects. Plants 8: 190 10.3390/plants8070190 31248005 PMC6681211

[24] Cao L, Jin X, Zhang Y (2019) Melatonin confers drought stress tolerance in soybean (*Glycine max* L.) by modulating photosynthesis, osmolytes, and reactive oxygen metabolism. Photosynthetica 57: 812–819.

[25] Kim J, Woo HR, Nam HG (2016) Toward systems understanding of leaf senescence: an integrated multi-omics perspective on leaf senescence research. Molecular plant 9: 813–825. 10.1016/j.molp.2016.04.017 27174403

[26] Gupta M, Bhaskar PB, Sriram S, Wang P-H (2017) Integration of omics approaches to understand oil/protein content during seed development in oilseed crops. Plant cell reports 36: 637–652. 10.1007/s00299-016-2064-1 27796489

[27] Meena KK, Sorty AM, Bitla UM, Choudhary K, Gupta P, et al (2017) Abiotic stress responses and microbe-mediated mitigation in plants: the omics strategies. Frontiers in plant science 8: 172 10.3389/fpls.2017.00172 28232845 PMC5299014

[28] Hu W-h, Yan X-h, Xiao Y-a, Zeng J-j, Qi H-j, et al (2013) 24-Epibrassinosteroid alleviate drought-induced inhibition of photosynthesis in Capsicum annuum. Scientia Horticulturae 150: 232–237.

[29] Lei Y, Xu Y, Hettenhausen C, Lu C, Shen G, et al (2018) Comparative analysis of alfalfa (*Medicago sativa* L.) leaf transcriptomes reveals genotype-specific salt tolerance mechanisms. BMC plant biology 18: 35 10.1186/s12870-018-1250-4 29448940 PMC5815232

[30] Kim D, Langmead B, Salzberg SL (2015) HISAT: a fast spliced aligner with low memory requirements. Nature methods 12: 357 10.1038/nmeth.3317 25751142 PMC4655817

[31] Anders S, Pyl PT, Huber W (2015) HTSeq—a Python framework to work with high-throughput sequencing data. Bioinformatics 31: 166–169. 10.1093/bioinformatics/btu638 25260700 PMC4287950

[32] Pertea M, Pertea GM, Antonescu CM, Chang T-C, Mendell JT, et al (2015) StringTie enables improved reconstruction of a transcriptome from RNA-seq reads. Nature biotechnology 33: 290 10.1038/nbt.3122 25690850 PMC4643835

[33] Anders S, Huber W (2010) Differential expression analysis for sequence count data. Genome biology 11: R106 10.1186/gb-2010-11-10-r106 20979621 PMC3218662

[34] Yu G, Wang L-G, Han Y, He Q-Y (2012) clusterProfiler: an R package for comparing biological themes among gene clusters. Omics: a journal of integrative biology 16: 284–287. 10.1089/omi.2011.0118 22455463 PMC3339379

[35] Camacho C, Coulouris G, Avagyan V, Ma N, Papadopoulos J, et al (2009) BLAST+: architecture and applications. BMC bioinformatics 10: 421 10.1186/1471-2105-10-421 20003500 PMC2803857

[36] Khan WA, Hou X, Han K, Khan N, Dong H, et al (2018) Lipidomic study reveals the effect of morphological variation and other metabolite interactions on the lipid composition in various cultivars of Bok choy. Biochemical and biophysical research communications 506: 755–764. 10.1016/j.bbrc.2018.04.112 29673595

[37] LIVAK (2001) Analysis of relative gene expression data using real-time quantitative PCR and the 2 (-Delta Delta C (T)) Method. Methods 25: 402–408. 10.1006/meth.2001.1262 11846609

[38] Manavalan LP, Guttikonda SK, Phan Tran L-S, Nguyen HT (2009) Physiological and molecular approaches to improve drought resistance in soybean. Plant and Cell Physiology 50: 1260–1276. 10.1093/pcp/pcp082 19546148

[39] Dubbels R, Reiter RJ, Klenke E, Goebel A, Schnakenberg E, et al (1995) Melatonin in edible plants identified by radioimmunoassay and by high performance liquid chromatography‐mass spectrometry. Journal of pineal research 18: 28–31. 10.1111/j.1600-079x.1995.tb00136.x 7776176

[40] Li C, Tan D-X, Liang D, Chang C, Jia D, et al (2014) Melatonin mediates the regulation of ABA metabolism, free-radical scavenging, and stomatal behaviour in two Malus species under drought stress. Journal of Experimental Botany 66: 669–680. 10.1093/jxb/eru476 25481689

[41] Meng JF, Xu TF, Wang ZZ, Fang YL, Xi ZM, et al (2014) The ameliorative effects of exogenous melatonin on grape cuttings under water‐deficient stress: antioxidant metabolites, leaf anatomy, and chloroplast morphology. Journal of Pineal Research 57: 200–212. 10.1111/jpi.12159 25039750

[42] Zhang N, Zhao B, Zhang HJ, Weeda S, Yang C, et al (2013) Melatonin promotes water‐stress tolerance, lateral root formation, and seed germination in cucumber (Cucumis sativus L.). Journal of Pineal Research 54: 15–23. 10.1111/j.1600-079X.2012.01015.x 22747917

[43] Liu J, Wang W, Wang L, Sun Y (2015) Exogenous melatonin improves seedling health index and drought tolerance in tomato. Plant growth regulation 77: 317–326.

[44] Ye J, Wang S, Deng X, Yin L, Xiong B, et al (2016) Melatonin increased maize (Zea mays L.) seedling drought tolerance by alleviating drought-induced photosynthetic inhibition and oxidative damage. Acta physiologiae plantarum 38: 48.

[45] Akula R, Ravishankar GA (2011) Influence of abiotic stress signals on secondary metabolites in plants. Plant signaling & behavior 6: 1720–1731. 22041989 10.4161/psb.6.11.17613 PMC3329344

[46] Dixon RA, Paiva NL (1995) Stress-induced phenylpropanoid metabolism. The plant cell 7: 1085 10.1105/tpc.7.7.1085 12242399 PMC160915

[47] Bettaieb I, Hamrouni-Sellami I, Bourgou S, Limam F, Marzouk B (2011) Drought effects on polyphenol composition and antioxidant activities in aerial parts of Salvia officinalis L. Acta Physiologiae Plantarum 33: 1103–1111.

[48] Ma D, Sun D, Wang C, Li Y, Guo T (2014) Expression of flavonoid biosynthesis genes and accumulation of flavonoid in wheat leaves in response to drought stress. Plant physiology and biochemistry 80: 60–66. 10.1016/j.plaphy.2014.03.024 24727789

[49] Guidi L, Degl'Innocenti E, Remorini D, Massai R, Tattini M (2008) Interactions of water stress and solar irradiance on the physiology and biochemistry of Ligustrum vulgare. Tree Physiology 28: 873–883. 10.1093/treephys/28.6.873 18381268

[50] Yuan Y, Liu Y, Wu C, Chen S, Wang Z, et al (2012) Water deficit affected flavonoid accumulation by regulating hormone metabolism in Scutellaria baicalensis Georgi roots. PloS one 7: e42946 10.1371/journal.pone.0042946 23077481 PMC3471899

[51] Ithal N, Reddy AR (2004) Rice flavonoid pathway genes, OsDfr and OsAns, are induced by dehydration, high salt and ABA, and contain stress responsive promoter elements that interact with the transcription activator, OsC1-MYB. Plant Science 166: 1505–1513.

[52] Jaakola L, Hohtola A (2010) Effect of latitude on flavonoid biosynthesis in plants. Plant, cell & environment 33: 1239–1247. 10.1111/j.1365-3040.2010.02154.x 20374534

[53] Naghizadeh M, Kabiri R, Hatami A, Oloumi H, Nasibi F, et al (2019) Exogenous application of melatonin mitigates the adverse effects of drought stress on morpho-physiological traits and secondary metabolites in Moldavian balm (Dracocephalum moldavica). Physiology and Molecular Biology of Plants 25: 881–894. 10.1007/s12298-019-00674-4 31402815 PMC6656836

[54] Liang D, Shen Y, Ni Z, Wang Q, Lei Z, et al (2018) Exogenous melatonin application delays senescence of kiwifruit leaves by regulating the antioxidant capacity and biosynthesis of flavonoids. Frontiers in plant science 9: 426 10.3389/fpls.2018.00426 29675031 PMC5896581

[55] Agati G, Tattini M (2010) Multiple functional roles of flavonoids in photoprotection. New Phytologist 186: 786–793. 10.1111/j.1469-8137.2010.03269.x 20569414

[56] Gamel RE, Elsayed A, Bashasha J, Haroun S (2017) Priming tomato cultivars in β-sitosterol or gibberellic acid improves tolerance for temperature stress. Int J Bot 13: 1–14.

[57] Abu-Muriefah SS (2015) Effect of sitosterol on growth, metabolism and protein pattern of pepper (Capsicum annuum L) plants grown under salt stress conditions. International Journal of Agriculture and Crop Sciences 8: 94.

[58] Fawzia AE, Ashraf AE, Samia AH, Hend AE Β-Sitosterol Ameliorates The Chemical Constituents Of Sunflower (Helianthus Annuus L.) Plants, Grown Under Saline Condition.

[59] Li Z, Cheng B, Yong B, Liu T, Peng Y, et al (2019) Metabolomics and physiological analyses reveal β-sitosterol as an important plant growth regulator inducing tolerance to water stress in white clover. Planta: 1–14. 10.1007/s00425-019-03277-1 31542810

