# Supplementary material for: Transcriptomic and metabolomic profiling of melatonin treated soybean (Glycine max L.) under drought stress during grain filling period through regulation of secondary metabolite biosynthesis pathways
Source: PLoS One. 2020 Oct 30;15(10):e0239701. doi: 10.1371/journal.pone.0239701 (PMC7598510; doi:10.1371/journal.pone.0239701)
Supplement: S1 Table — (DOCX) [file pone.0239701.s001.docx]

**S1 Table The growth temperature**

| Date | Temperature (°C) |
| --- | --- |
| August 10^th^ | 18.70 |
| August 11^th^ | 19.80 |
| August 12^th^ | 20.60 |
| August 13^th^ | 21.80 |
| August 14^th^ | 23.50 |
| August 15^th^ | 23.90 |
| August 16^th^ | 19.40 |
| August 17^th^ | 18.00 |
| August 18^th^ | 18.80 |
| August 19^th^ | 19.70 |
| August 20^th^ | 20.80 |
| August 21^th^ | 19.50 |
| August 22^th^ | 18.30 |
| August 23^th^ | 19.40 |
| August 24^th^ | 20.50 |
| August 25^th^ | 21.00 |
| August 26^th^ | 20.40 |
