# Supplementary material for: Transcriptomic and metabolomic profiling of melatonin treated soybean (Glycine max L.) under drought stress during grain filling period through regulation of secondary metabolite biosynthesis pathways
Source: PLoS One. 2020 Oct 30;15(10):e0239701. doi: 10.1371/journal.pone.0239701 (PMC7598510; doi:10.1371/journal.pone.0239701)
Supplement: S2 Table — (DOCX) [file pone.0239701.s002.docx]

**S2 Table** Primer sequences of DEGs for RT-qPCR.

| Gene | Encoded protein | Gene accession | Primer sequence (5'-3') |
| --- | --- | --- | --- |
| 1.Glyma.18G267800 | isoflavone-7-O-methyltransferase | LOC100812688 | F: TATTCCCAAGGCCTGTGCAG |
|  |  |  | R: GAACCCTGCTTCCACGAAGA |
| 2.Glyma.09G049300 | CYP81E1_7 | I2'H | F: TCGCCGTTGTCATTTCCTCA |
|  |  |  | R: ACCTTTTCGTCTCGTCGCTT |
| 3.Glyma.09G269400 | vestitone reductase | LOC102659875 | F: CTGCTGTTTATTGGCAGGGC |
|  |  |  | R: CGAGAGGCACCAAGTGGATT |
| 4.Glyma.06G239500 | UGT72E | LOC100792386 | F: AAGAGATAGCGCGGATGGTG |
|  |  |  | R: ATCGCTCATCAGCTAGCACC |
| 5.Glyma.19G132900 | lysosomal acid lipase | SDP1-3 | F: TTGGACAAGATCTGGTGGGC |
|  |  |  | R: ACCCTGTTGCCAATTTCCCT |
| 6.Glyma.13G217400 | sterol 22-desaturase | LOC100787616 | F: GGTCCGAGACCCAACCAATT |
|  |  |  | R: TGTAAAGTTGGGGGCGATCC |
| 7. GAPDH1 |  |  | F:GGAGTGTTCACTGACAAG |
|  |  |  | R: CAAGCGGAGCAAGACAGTTG |
