# Supplementary figures and images for: Transcriptomic and metabolomic profiling of melatonin treated soybean (Glycine max L.) under drought stress during grain filling period through regulation of secondary metabolite biosynthesis pathways
Source: PLoS One. 2020 Oct 30;15(10):e0239701. doi: 10.1371/journal.pone.0239701 (PMC7598510; doi:10.1371/journal.pone.0239701)

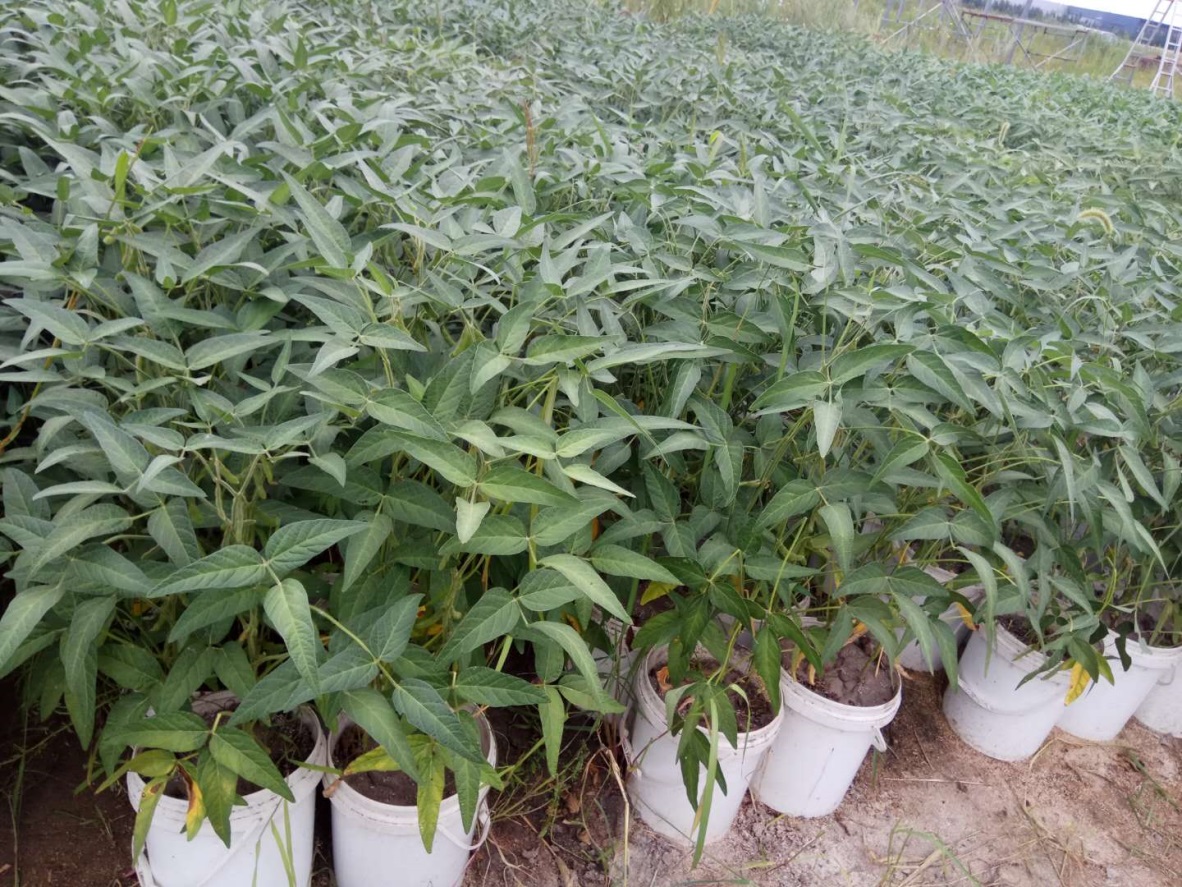


**S1 Fig** The photo of potted plants during grain filling stage.

Supplement: S1 Fig — (DOCX) [file pone.0239701.s003.docx]

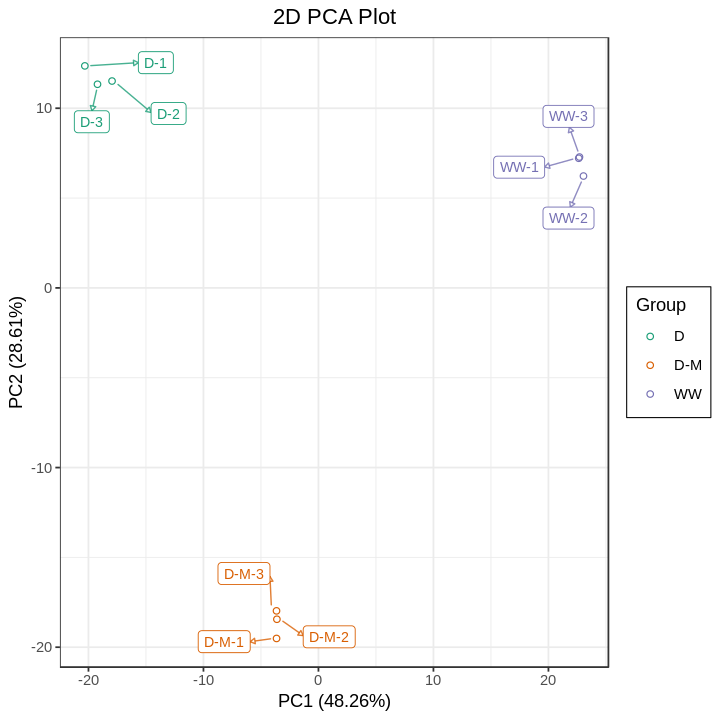


**S6 Fig** PCA analysis of metabonomics.

Supplement: S6 Fig — (DOCX) [file pone.0239701.s008.docx]
