# Supplementary material for: Transcriptomic and metabolomic profiling of melatonin treated soybean (Glycine max L.) under drought stress during grain filling period through regulation of secondary metabolite biosynthesis pathways
Source: PLoS One. 2020 Oct 30;15(10):e0239701. doi: 10.1371/journal.pone.0239701 (PMC7598510; doi:10.1371/journal.pone.0239701)
Supplement: S3 Fig — Black dots represent genes without significant differential expression; red and green dots denote significantly up-regulated and down-regulated genes respectively in the WW/D and D/D-M comparisons. (DOCX) [file pone.0239701.s005.docx]

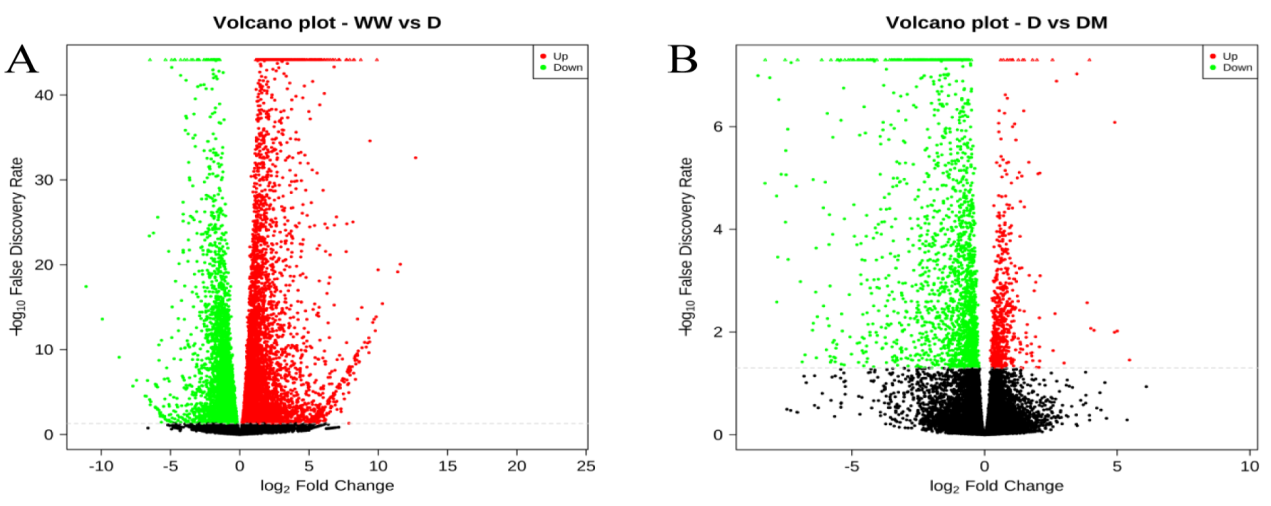


**S3 Fig** Volcano plots of DEGs in (A) WW/D and (B) D/D-M comparisons. Black dots represent genes without significant differential expression; red and green dots denote significantly up-regulated and down-regulated genes respectively in the WW/D and D/D-M comparisons.
