# Supplementary material for: Transcriptomic and metabolomic profiling of melatonin treated soybean (Glycine max L.) under drought stress during grain filling period through regulation of secondary metabolite biosynthesis pathways
Source: PLoS One. 2020 Oct 30;15(10):e0239701. doi: 10.1371/journal.pone.0239701 (PMC7598510; doi:10.1371/journal.pone.0239701)
Supplement: S4 Fig — (A) The Venn diagram shows the overlapped DEGs between the WW/D and D/D-M comparisons. (B) Heat maps of the overlaped DEGs between the WW/D and D/D-M comparisons. (DOCX) [file pone.0239701.s006.docx]

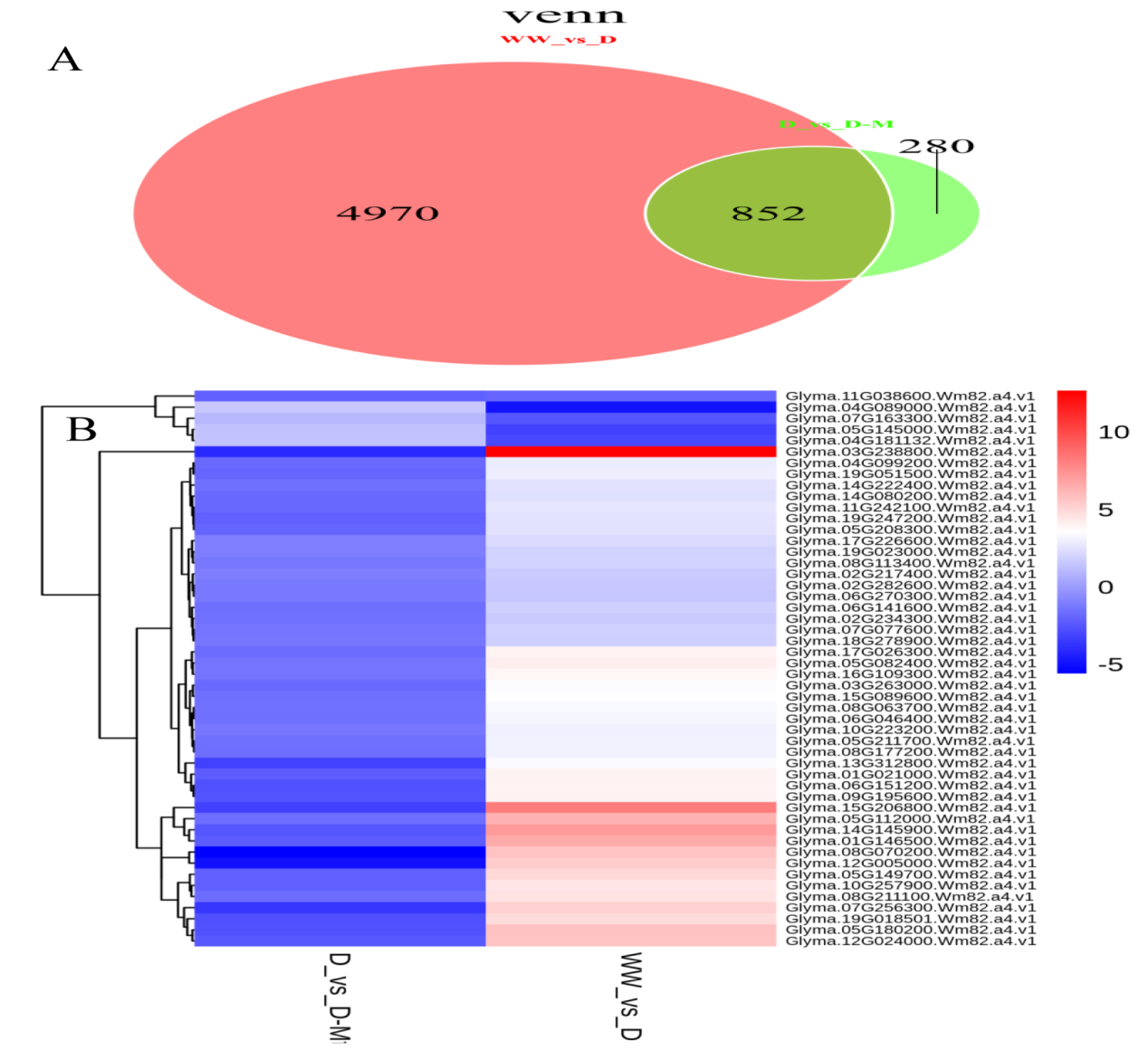


**S4 Fig** Drought stress and exogenous melatonin treatment affect alterations in transcriptome. (A) The Venn diagram shows the overlapped DEGs between the WW/D and D/D-M comparisons. (B) Heat maps of the overlaped DEGs between the WW/D and D/D-M comparisons.
