# Supplementary material for: Transcriptomic and metabolomic profiling of melatonin treated soybean (Glycine max L.) under drought stress during grain filling period through regulation of secondary metabolite biosynthesis pathways
Source: PLoS One. 2020 Oct 30;15(10):e0239701. doi: 10.1371/journal.pone.0239701 (PMC7598510; doi:10.1371/journal.pone.0239701)
Supplement: S5 Fig — (A) GO analysis of DEGs in the WW/D comparison; and (B) GO analysis of DEGs in the D/D-M comparison. (DOCX) [file pone.0239701.s007.docx]

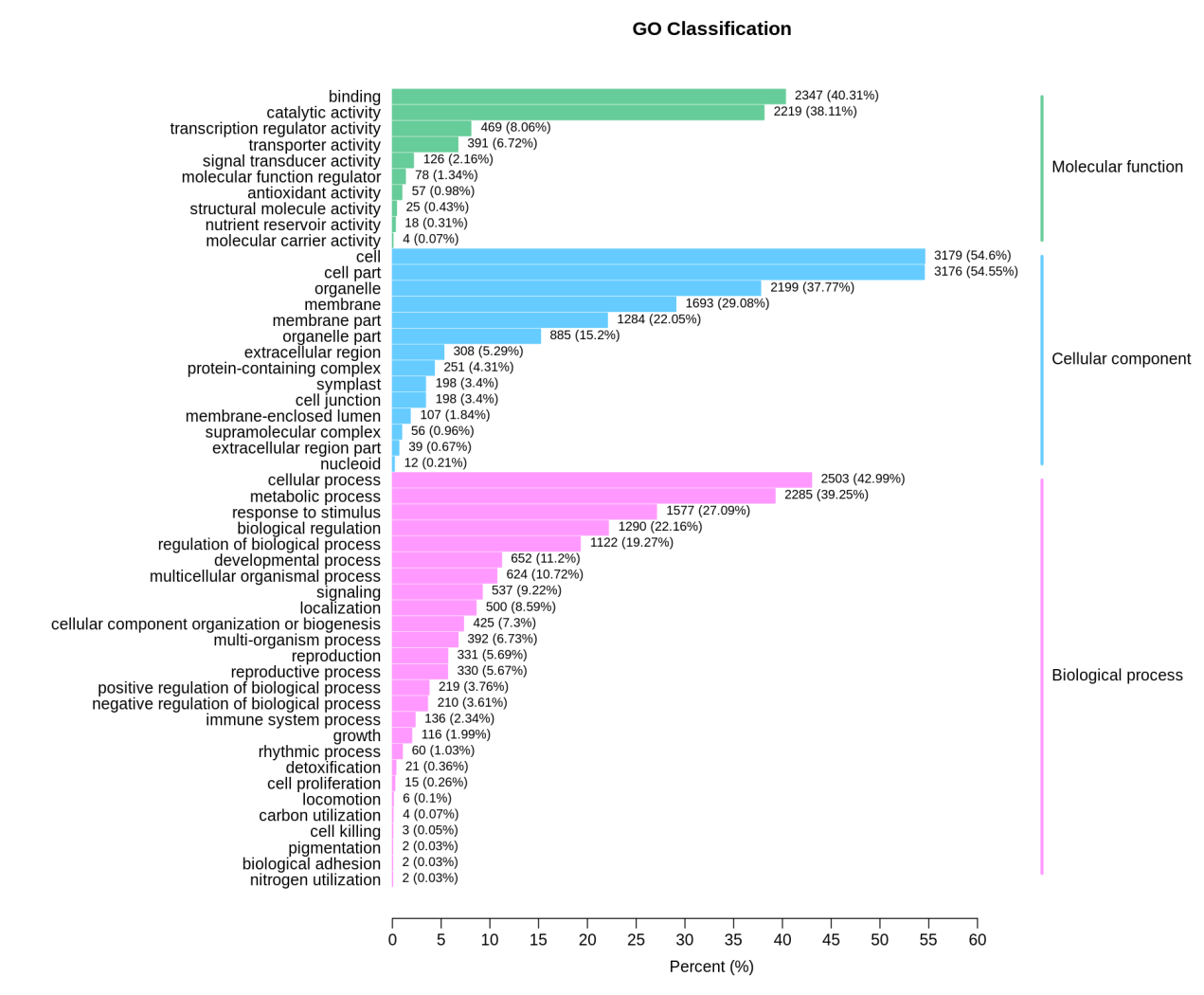


A


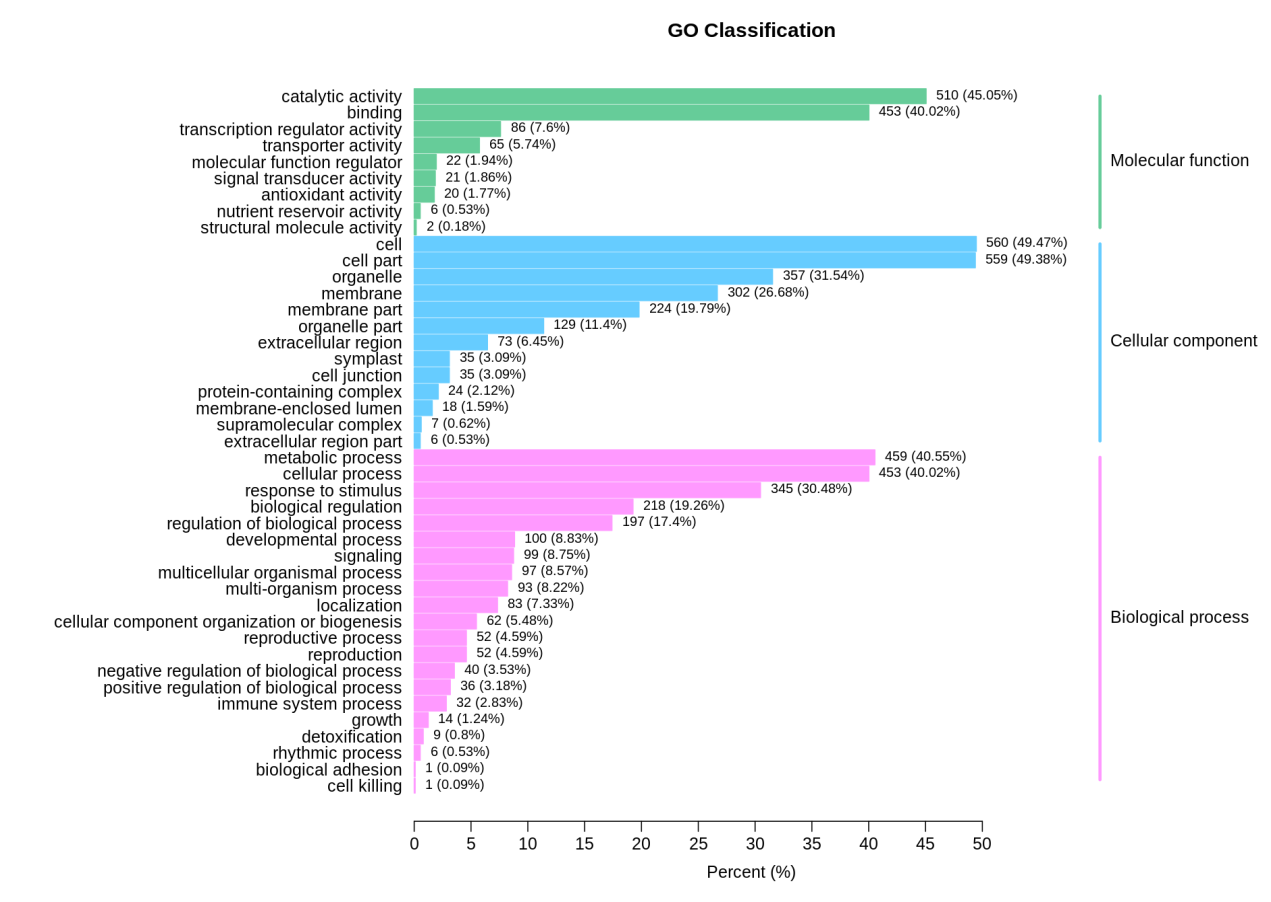


B

**S5 Fig** Gene Ontology (GO) classifications of differentially expressed genes (DEGs). (A) GO analysis of DEGs in the WW/D comparison; and (B) GO analysis of DEGs in the D/D-M comparison.
