# Supplementary material for: Transcriptomic and metabolomic profiling of melatonin treated soybean (Glycine max L.) under drought stress during grain filling period through regulation of secondary metabolite biosynthesis pathways
Source: PLoS One. 2020 Oct 30;15(10):e0239701. doi: 10.1371/journal.pone.0239701 (PMC7598510; doi:10.1371/journal.pone.0239701)
Supplement: S7 Fig — (A) The Venn diagram shows the overlapped differentially-accumulated metabolites between the WW/D and D/D-M comparisons, (B) Histogram of the differentially-accumulated metabolites in the WW/D comparison, and (C) Histogram of the differentially-accumulated metabolites in the D/D-M comparison. (DOCX) [file pone.0239701.s009.docx]

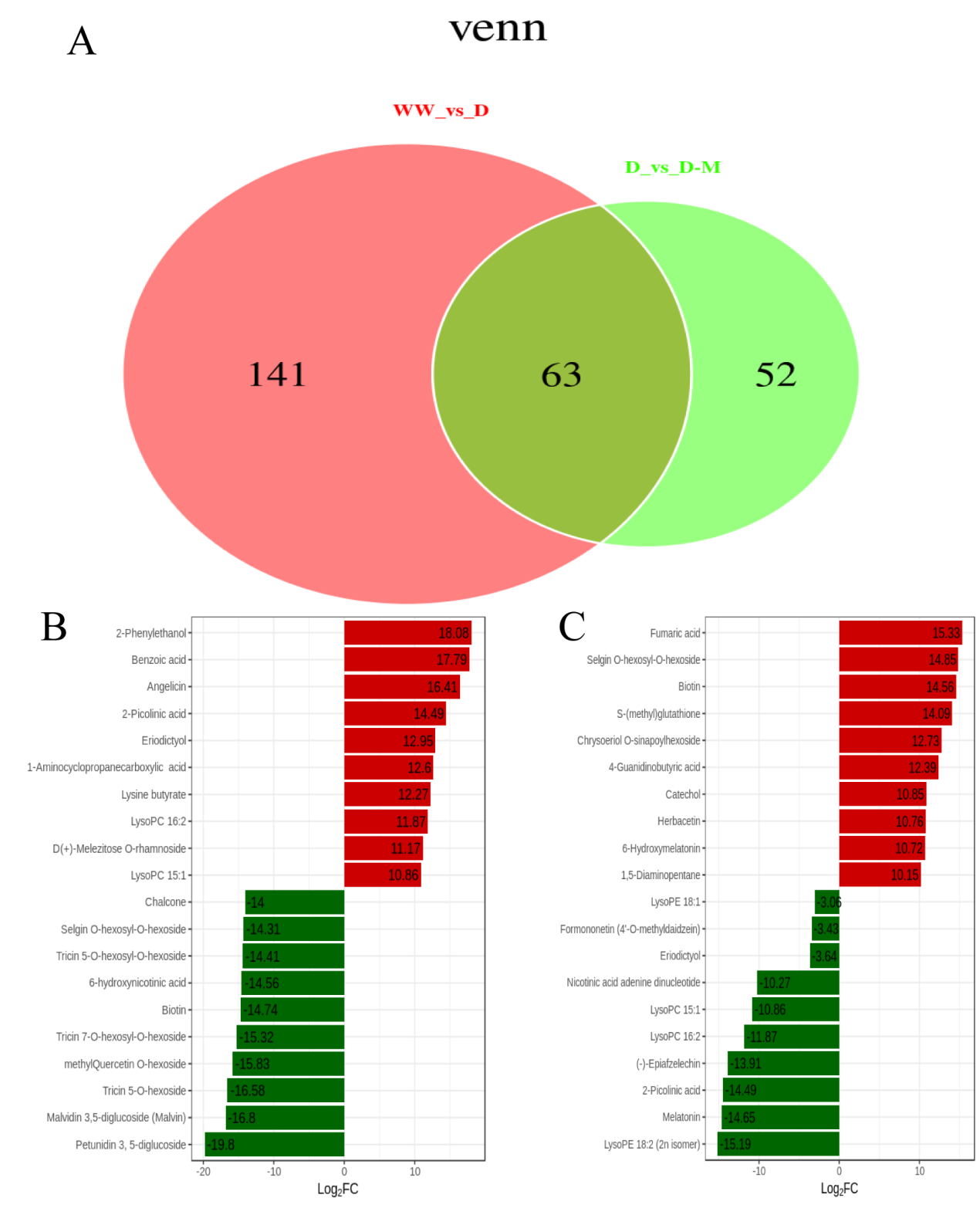


**S7 Fig** Drought stress and exogenous melatonin treatment affect alterations in metabolome. (A) The Venn diagram shows the overlapped differentially-accumulated metabolites between the WW/D and D/D-M comparisons, (B) Histogram of the differentially-accumulated metabolites in the WW/D comparison, and (C) Histogram of the differentially-accumulated metabolites in the D/D-M comparison.
