# Supplementary material for: Transcriptomic and metabolomic profiling of melatonin treated soybean (Glycine max L.) under drought stress during grain filling period through regulation of secondary metabolite biosynthesis pathways
Source: PLoS One. 2020 Oct 30;15(10):e0239701. doi: 10.1371/journal.pone.0239701 (PMC7598510; doi:10.1371/journal.pone.0239701)
Supplement: S8 Fig — (A) WW/D comparison and (B) D/D-M comparison. (DOCX) [file pone.0239701.s010.docx]

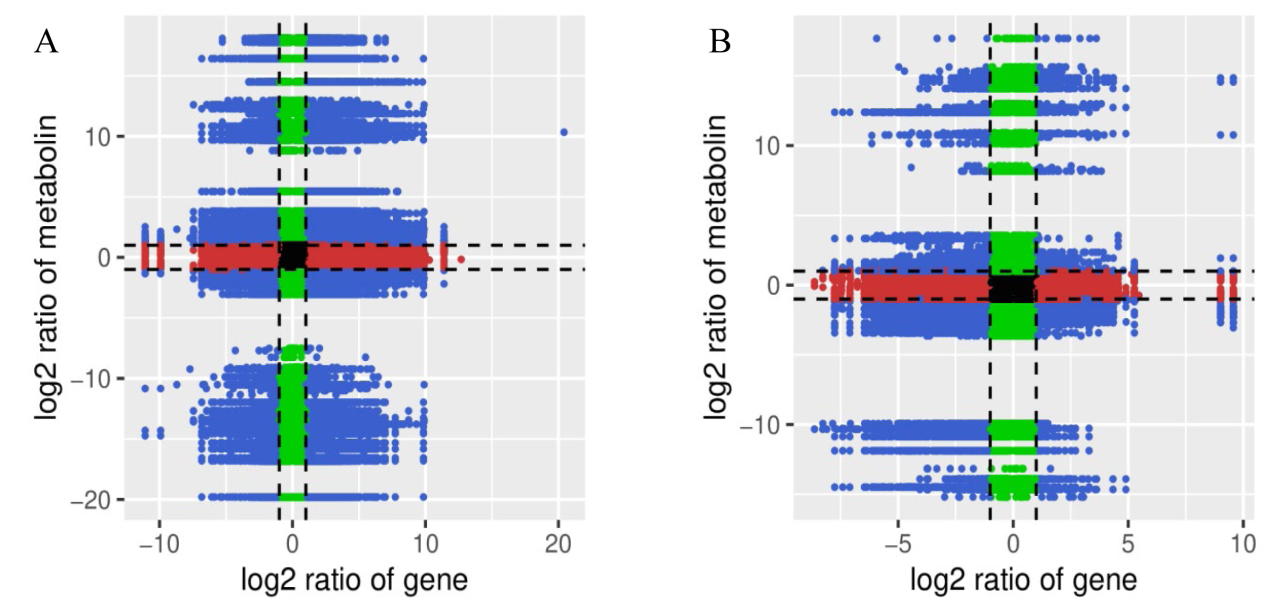


**Figure S8** Conjoint analysis between transcriptomic and metabolomic changes by nine-quadrants. (A) WW/D comparison and (B) D/D-M comparison.
