# Supplementary material for: Transcriptomic and metabolomic profiling of melatonin treated soybean (Glycine max L.) under drought stress during grain filling period through regulation of secondary metabolite biosynthesis pathways
Source: PLoS One. 2020 Oct 30;15(10):e0239701. doi: 10.1371/journal.pone.0239701 (PMC7598510; doi:10.1371/journal.pone.0239701)
Supplement: S11 Fig — (A) HPLC chromatogram of β-sitosterol in standard solution; (B) HPLC chromatogram of β-sitosterol in the leaf of soybean in the WW group; (C) HPLC chromatogram of β-sitosterol in the leaf of soybean in the D group; and (D) HPLC chromatogram of β-sitosterol in the leaf of soybean in the D-M group. (DOCX) [file pone.0239701.s013.docx]

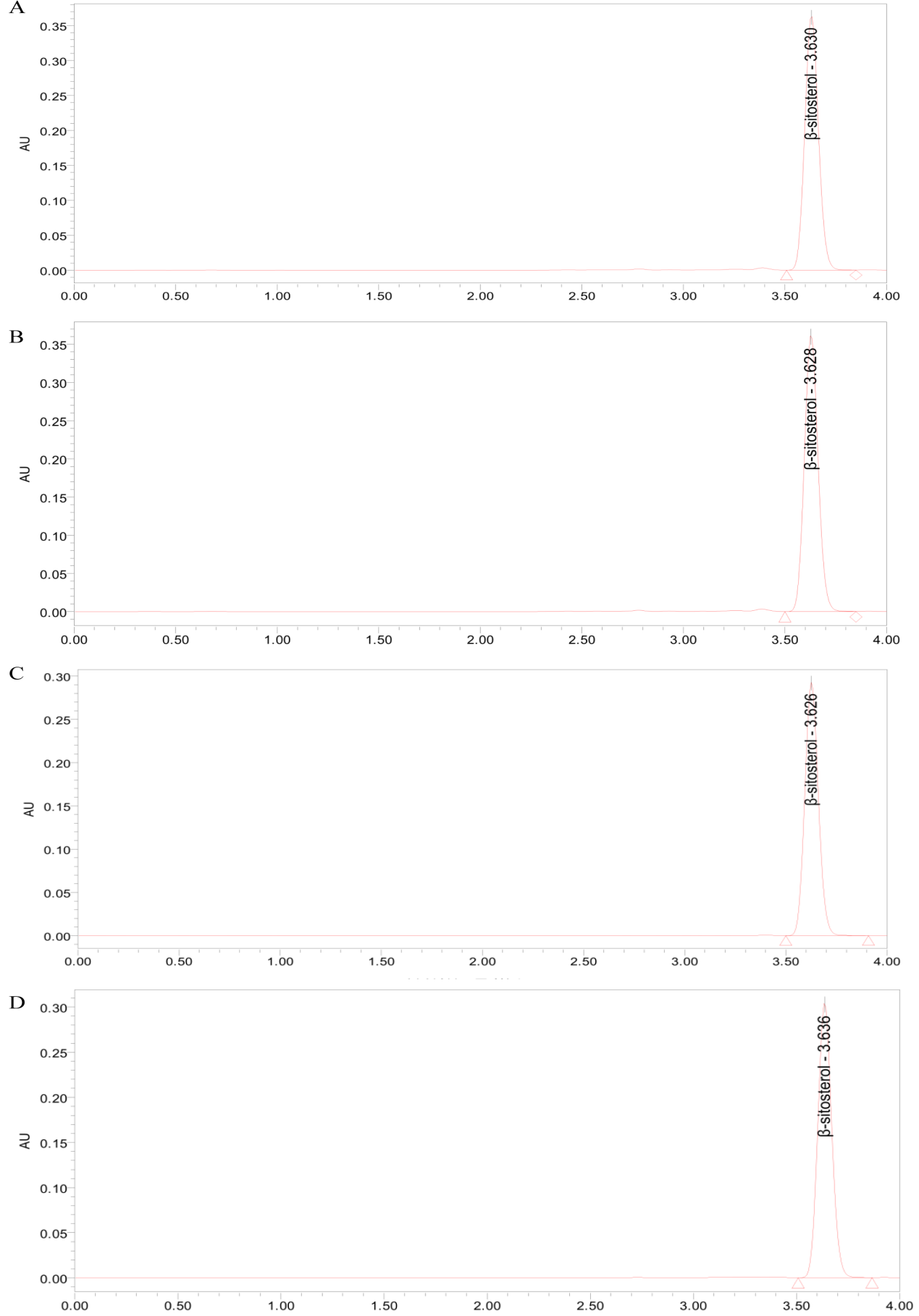


**S11 Fig** The representative HPLC chromatograms of β-sitosterol detecting at wavelength of 205 nm. (A) HPLC chromatogram of β-sitosterol in standard solution; (B) HPLC chromatogram of β-sitosterol in the leaf of soybean in the WW group; (C) HPLC chromatogram of β-sitosterol in the leaf of soybean in the D group; and (D) HPLC chromatogram of β-sitosterol in the leaf of soybean in the D-M group.
